# Intracellular Selection of Theophylline-Sensitive Hammerhead Aptazyme

**DOI:** 10.1016/j.omtn.2020.03.001

**Published:** 2020-03-13

**Authors:** Qinlin Pu, Shan Zhou, Xin Huang, Yi Yuan, Feng Du, Juan Dong, Gangyi Chen, Xin Cui, Zhuo Tang

**Affiliations:** 1 Natural Products Research Center, Chengdu Institution of Biology, Chinese Academy of Science, Chengdu 610041, P.R. China; 2 University of Chinese Academy of Sciences, Beijing 10049, P.R. China

## Abstract

Hammerhead ribozyme-based aptazyme (HHAz), inheriting the advantages of small size and high efficiency from the RNA-cleaving ribozyme and the specific recognition ability of aptamers to specific targets, exhibits the huge potential to be a transgene expression regulator. Herein, we report a selection strategy for HHAz by using a toxin protein IbsC as the reporter to offer a positive phenotype, thus realizing an easy-operating, time- and labor-saving selection of HHAz variants with desired properties. Based on this strategy, we obtained a new HHAz (TAP-1), which could react sensitively toward the extracellular regulatory molecule, theophylline, both in prokaryotic and eukaryotic systems. With fluorescent protein reporter, the intracellular switching efficiencies of TAP-1 and other reported theophylline-dependent HHAzs has been quantitatively evaluated, showing that TAP-1 not only exhibits the best downregulating ability at high concentration of theophylline but also maintains high activity with 0.1 mM theophylline, which is a safe concentration in the human body.

## Graphical Abstract

**Figure:**
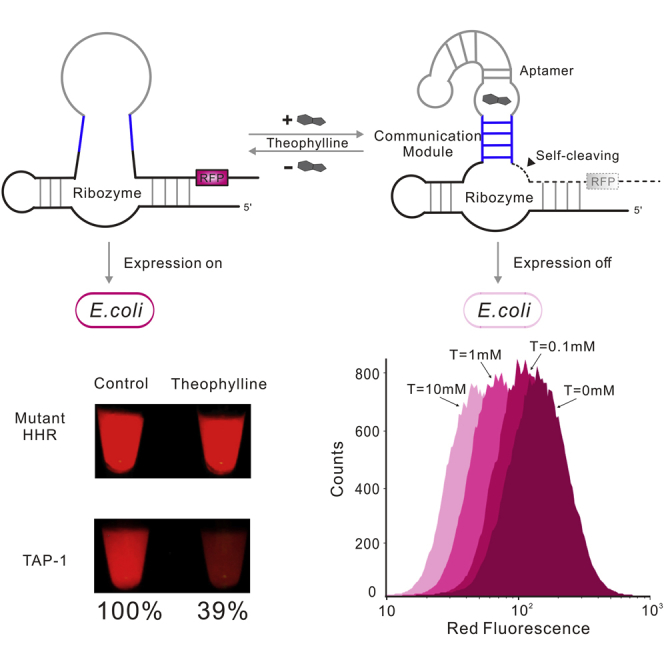

## Introduction

**Figure 1:**
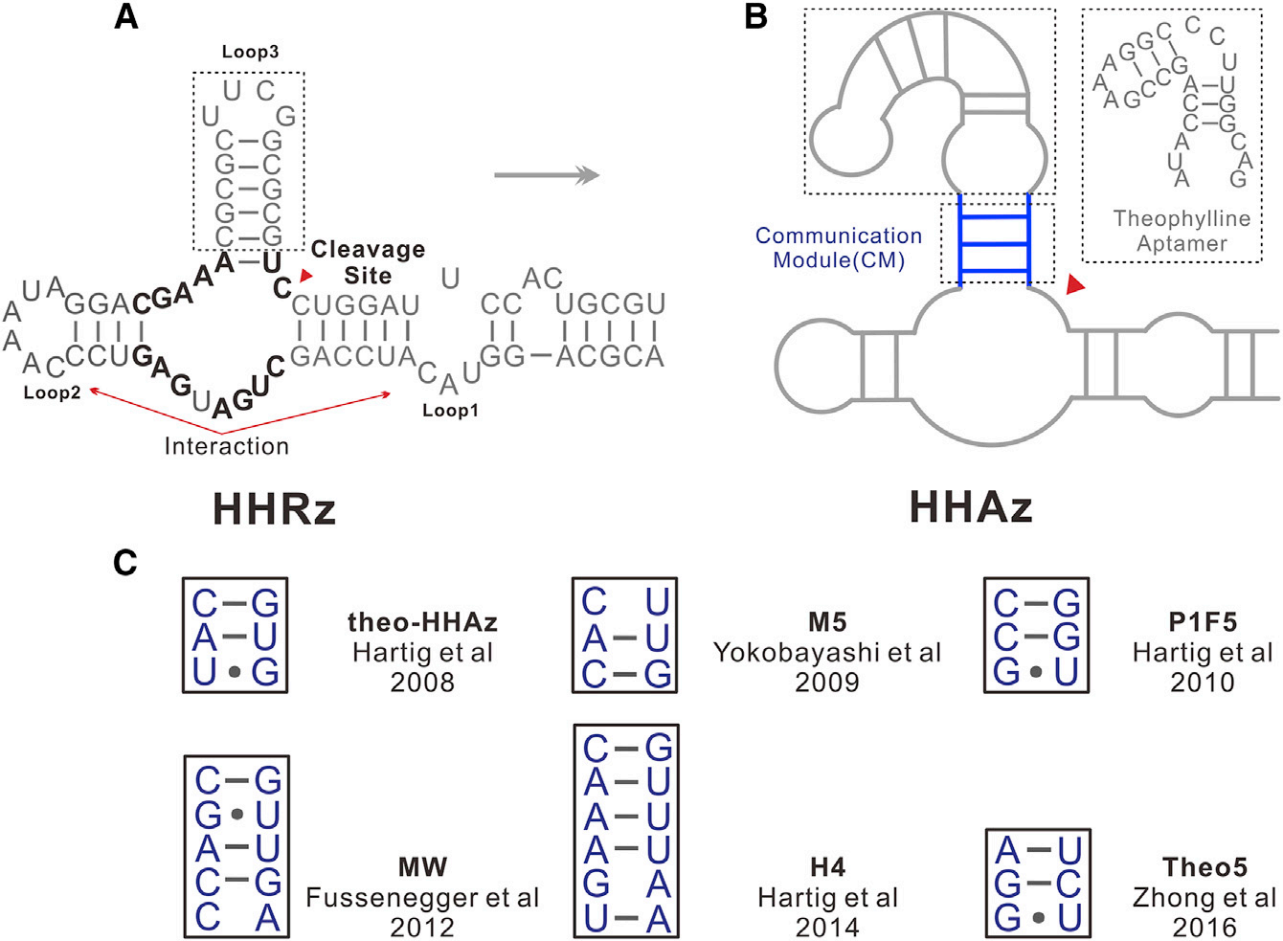
Illustration of Secondary Structures (A) The secondary structure of a hammerhead ribozyme from *Schistosoma Mansoni*. (B) The illustration of a theophylline-dependent hammerhead aptazyme. (C) A list of communication modules of reported theophylline-dependent HHAzs that have been mentioned for modulation ability comparison in this report.

Riboswitches, first termed in 2002,^1^ are *cis*-acting RNA structures that modulate gene expression upon specific ligand binding without protein engagement. Natural riboswitches, though they commonly exist *in vivo*, are confined to bind with intracellular molecules to regulate metabolic-related gene expression, whereas purposeful gene expression control with exogenous ligands is largely dependent on the development of artificial riboswitches.^2^ It is well accepted that riboswitches are composed of an aptamer domain and an expression platform. The aptamer domain is for specific recognizing of ligands; the expression platform is for converting the aptamer-ligand recognition into the regulation of the gene expression.^3^ Riboswitches regulate gene expression commonly by either allosteric or catalytic principles.^4^ RNA-cleaving ribozymes are often utilized to construct artificial riboswitches. Among diverse candidates, hammerhead ribozyme (HHRz, see Figure 1A) stands out for its advantages of small size, simple structure, and high modularity, and it has already been utilized as a safe and highly efficient molecular tool for knocking down of gene expression.^5^ Moreover, HHRz-based riboswitch is also termed as hammerhead aptazyme (HHAz), which is HHRz appended with an aptamer domain to modulate its self-cleavage.^6^ Owing to the development of the systematic evolution of ligands by exponential enrichment (SELEX),^7^ RNA aptamers are massively described, which makes it theoretically possible to develop a specific RNA aptamer for any exogenous ligand. For now, only a few RNA aptamers obtained through SELEX have been verified to maintain their binding activity intracellularly, such as tetracycline aptamer,^8^ neomycin aptamer,^9^ tobramycin aptamer,^10^ and theophylline aptamer.^11^ On account of the possibility of future applications in gene therapy, gene expression regulation with aminoglycoside antibiotic ligands, such as tetracycline and neomycin, is possible to pose potential threats of antibiotic abuse toward public health. But, theophylline, a common medicine for the treatment of chronic asthma, is a safer ligand to use. Besides, a safe amount of theophylline is easily accessible in common beverages, such as tea.

The binding of certain ligands either promotes or hinders the self-cleavage of HHAzs. The promotion of self-cleavage is mainly realized by the formation of the stable catalytic core of HHRz,^12^ while various mechanisms could be utilized to hinder the self-cleavage.^13^ Among these HHAz variants, the aptamer domain is generally appended to HHRz through a communication module (CM; see Figure 1B). A CM is expected to be a proper transmitter that transmits the ligand-binding signal to the catalytic core of HHRz through a conformational change.^14^ A CM that is too flexible will trigger no catalytic cleavage even with a ligand binding. On the contrary, a CM that is too stable will cause self-cleavage without a ligand binding. Two different methods, rational design and intracellular selection, are employed to obtain variants with diverse CMs. The first artificial HHAz is constructed through rational design, and verified *in vitro* by Breaker et al.,^15^ while they only work extracellularly. Notably, many theophylline-dependent HHAz variants obtained through calculation-assisted rational design, are well functioned as regulation elements during the gene expression process in eukaryotic systems.^16^^,^^17^ However, the intracellular environment is so complicated that it is hard to predict with theoretical thermodynamic parameters. On the contrary, the intracellular selection is more suitable for directly obtaining HHAz variants that target intracellular applications. The strategy of intracellular selection is often engaging with fluorescence protein such as *eGFP* or *mCherry* as its reporter. What’s more, fluorescence-activated cell sorting (FACS) is utilized frequently among these intracellular selection strategies.^13^^,^^18^ During the selection process, host cell with a decreased or increased intensity of fluorescence signal, caused by self-cleavage of an aptazyme after ligand binding, is often selected with FACS. However, the fluorescence signal of single host cell is easily affected by unintentional factors. Besides, FACS-based intracellular selection is highly dependent on the equipment of high-speed cell sorting flow cytometer (FCM). Moreover, with the increasing selection library, the selection procedure becomes increasingly time and labor consuming, adding much inconvenience to the selection procedure. Therefore, a simple and easy-operating intracellular selection strategy is highly desirable.

In the recent two decades, many theophylline-dependent HHAz variants were developed through rational design and intracellular selection strategies^17^^,^^19^, ^20^, ^21^, ^22^, ^23^ (see Figure 1C), and these variants also achieved regulation capability in many fields. To exemplify, in 2008, theophylline-dependent HHAz was utilized to construct high-order cellular devices for information processing;^24^ in 2009, the process of RNA interference was realized to be conditionally controlled by theophylline-dependent HHAz variants;^20^ in 2010, it was realized to regulate the proliferation of mammalian T cell by using theophylline-dependent HHAz;^25^ in 2012, theophylline-dependent HHAz was applied to *in vivo* screening of caffeine demethylase enzyme;^26^ in 2017, the effectiveness of nuclease Cas9 was realized to be modulated by theophylline-dependent HHAz for off-targeting control;^27^ last but not least, in gene therapy, adenovirus-based gene expression and safety control is also involving the modulation of theophylline-dependent HHAz variants.^28^, ^29^, ^30^ When it comes to applying a certain theophylline-dependent HHAz variant, which one to choose becomes a question because no proper comparison is conducted. Besides, we identified a problem that the experimental concentration of theophylline is as high as 2–5 mM to realize certain regulation effects. However, as reported in clinical researches,^31^ more than 20 mg/L (around 0.11 mM) of theophylline in blood will cause many adverse effects of anorexia, nausea, and vomiting, sometimes seizure in adults. Therefore, to take the further application of theophylline-dependent-HHAz-based gene therapy into consideration, it is urgent to screen HHAz variants that react sensitively under the safe concentration of theophylline.

Herein, we report a selection strategy for HHAz by using a toxin protein IbsC as the reporter to offer a positive phenotype, which makes the selection of desired HHAz conveniently and efficiently. Based on this strategy, we obtained a new theophylline-dependent HHAz variant, which could react more sensitively toward theophylline with a wider concentrate range than the reported theophylline-dependent HHAz variants in both prokaryotic and eukaryotic systems.

## Results

### Selection Strategy Based on a Toxin Protein IbsC

**Figure 2:**
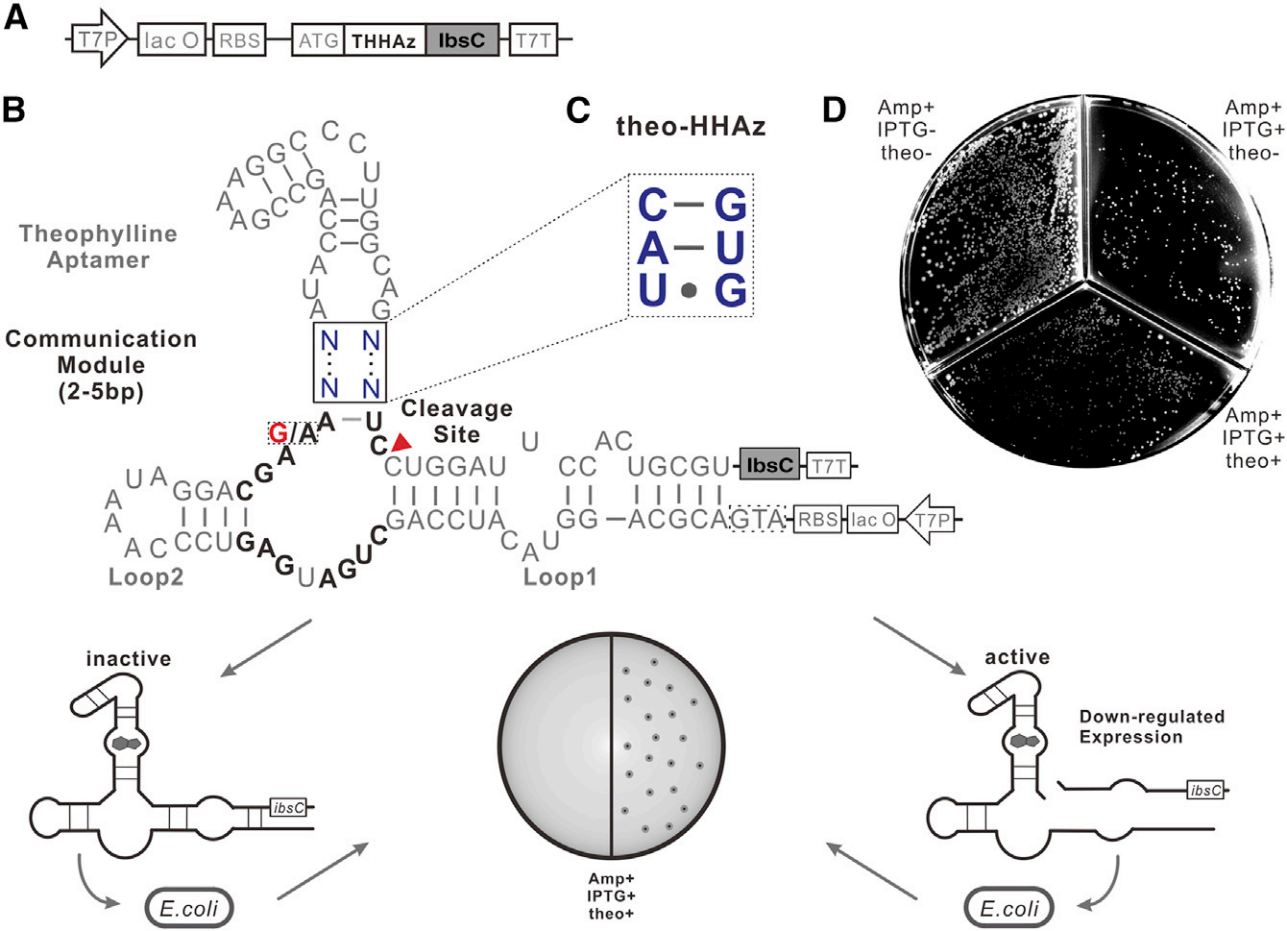
Selection Strategy (A) The illustration for construction of selection vector basing on toxin protein IbsC. The HHAz randomized library is designed to fusion express with IbsC at its N terminus. (B) The illustration of the selection strategy. The boxed blue N is the randomized CM domain. 2–5 base pairs of randomized sequence were designed into the CM domain for self-cleaving modulation. The selection plate was added with 100 μg/mL ampicillin, 1 mM IPTG, and 0.5 mM theophylline. (C) The illustration of CM domain of theo-HHAz. (D) The result of different viability for *E. coli* transferred with theo-HHAz inserted plasmid on the solid medium added with and without theophylline. The concentration of ampicillin is 100 μg/mL, IPTG is 1 mM, and theophylline is 1 mM.

In our intracellular selection strategy, *Escherichia coli* (*E. coli*) was chosen to harbor the randomized pool for intracellular selection, because every *E. coli* can only be transformed by one plasmid. Since the self-cleaving efficiency determines the phonotype of each host cell, the higher the self-cleaving efficiency, the less the corresponding protein expression, which makes it a negative signal for the given phenotype. The positive phenotype is more desirable than a negative signal since undesirable factors easily cause negative signals. IbsC^32^ is an *E. coli*-originated toxin protein. It is a highly hydrophobic peptide, which anchors in the inner membrane of the bacterium after expression.^33^ The overexpressed IbsC stagnates the growth status of *E. coli* by depolarizing the cell membrane and compromising the integrity of the cell envelope.^34^ Moreover, extensive mutations are highly tolerable for IbsC, and it is feasible to fusion express with other protein at its N terminus without losing its toxicity. For instance, IbsC was reported to fusion express with wild-type HHRz (HHRz) and inactivated HHR (HHRzm, harboring an A14 to G14 in the catalytic core, see Figure 2B) at the N terminus, which doesn’t compromise its toxicity^35^ (see also Figure S1). With these characteristics, the toxin protein IbsC is qualified for converting the negative signal, i.e., downregulation of IbsC, into a positive signal, i.e., colony growth on the solid plates.

As shown in Figure 2A, between the ribosomal binding site (RBS) and the open reading frame (ORF) of IbsC, the randomized pool of HHAz variants is inserted to fusion express with the toxin protein IbsC at its N terminus. They are both under the control of lacO operator. Besides, it has been reported that the interaction between Loop1 and Loop2 is the insurance of highly efficient self-cleavage for HHRz.^36^^,^^37^ Therefore, the theophylline aptamer is designed to replace Loop3, and link to the catalytic core of HHRz through a randomized CM, leaving the Loop1 and Loop2 interaction undisturbed (see Figure 2B). When the expression is induced, as long as an invalid HHAz structure in the random pool fails to cleave itself, the downstream toxin protein IbsC will be expressed. Consequently, no visible colony expressing the invalid structure will grow on the Petri dish added with isopropyl β-D-1-thiogalactopyranoside (IPTG) and theophylline. On the contrary, as long as an HHAz structure in the random pool cleaves itself efficiently upon binding theophylline, the toxin protein IbsC will fail to express, resulting in the survival of corresponding bacteria colony on the Petri dish added with IPTG and theophylline. After sequencing the transferred vector from the very harvested bacteria, the sequence of the efficient HHAz variant can be obtained.

Before selection, it is necessary to verify the feasibility of the strategy that theophylline can regulate the expression of IbsC, resulting in different survival rates between the solid medium plates added with and without theophylline. Therefore, theo-HHAz (Figure 2C), reported by Hartig et al.^19^ in 2008, was inserted to testify whether theophylline improves the self-cleavage efficiency of HHAz, downregulating the toxin protein IbsC. As shown in Figure 2D, *E. coli* transferred with theo-HHAz inserted plasmids exhibited different viability between the solid medium plates added with and without theophylline. This result verified the feasibility of our strategy.

### Intracellular Selection Based on Toxin Protein IbsC

**Figure 3:**
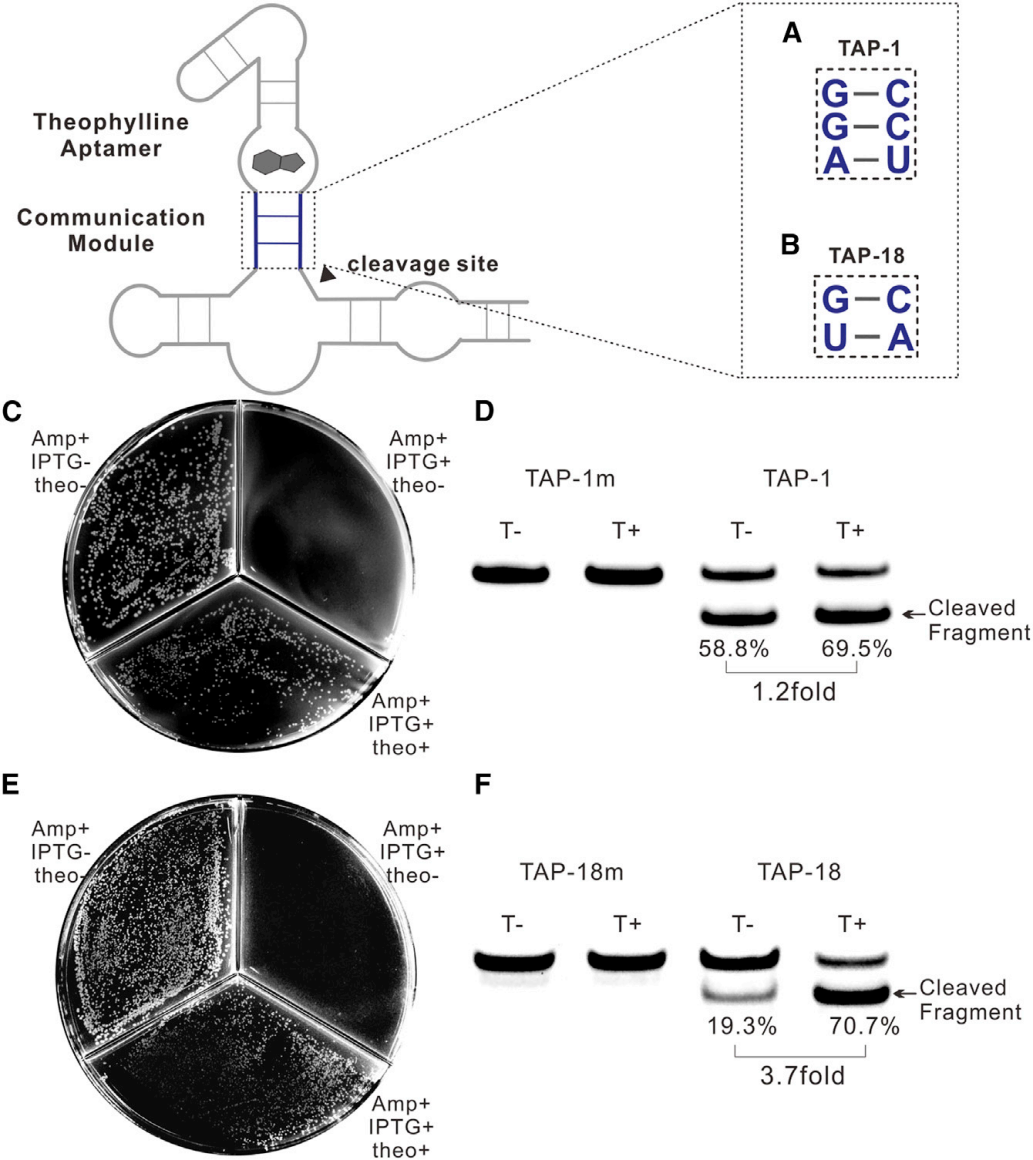
Selection Results (A) The illustration of CM domain of TAP-1. (B) The illustration of CM domain of TAP-18. (C) The result of different viability for *E. coli* transferred with TAP-1 inserted plasmid on the solid medium added with and without theophylline. The concentration of ampicillin is 100 μg/mL, IPTG is 1 mM, and theophylline is 1 mM. (D) PAGE analysis of FAM-labeled trans-cleaving result of TAP-1. TAP-1m was set as the negative control. The reaction is performed in 50 mM Tris-HCl (pH 7.5) buffer containing 10 mM MgCl_2_ at 37°C for 1 h with or without 1 mM theophylline. Different fraction of cleaved fragment was identified from the cleaving reactions with and without theophylline (data with error bar was supplemented in Figure S3). (E) The result of different viability for *E. coli* transferred with TAP-18 inserted plasmid on the solid medium added with and without theophylline. The concentration of ampicillin is 100 μg/mL, IPTG is 1 mM, and theophylline is 1 mM. (F) PAGE analysis of FAM-labeled *trans*-cleaving result of TAP-18. TAP-18m was set as the negative control. The reaction is performed in 50 mM Tris-HCl (pH 7.5) buffer containing 10 mM MgCl_2_ at 37°C for 1 h with or without 1 mM theophylline. Different fraction of cleaved fragment was identified from the cleaving reactions with and without theophylline (quantification data was supplemented in Figure S3).

Based on the toxic protein IbsC, we started a systematic investigation. 2–5 base pairs of randomized CM domain were constructed into the recombinant plasmids and transferred into *E. coli* (designed randomized sequences were displayed in Table S1). Then, the library was incubated on the solid Luria-Burtani (LB) plate with 100 μg/mL ampicillin, 1 mM IPTG, and theophylline. With the induction of IPTG, the fusion mRNA of HHAz variants and *ibsC* would be massively transcribed. Only when the ones efficiently cleave themselves after binding theophylline will they survive on the plates and grow into a visible colony. From the 10^7^ library, we harvested loads of colonies that have helped *E. coli* survive on the plate with 2 mM theophylline. To increase the selection pressure, we mixed the survived colonies again and then incubated them on the plates with 1 mM theophylline. The same procedure was repeated on the plates with 0.5 mM, 0.2 mM, and 0.1 mM in turn. In the end, we obtained 38 HHAz variants from the selection procedures (see Table S2). After reapplying the selected variants on the plates with and without theophylline at the same time, TAP-1 (Figure 3A) and TAP-18 (Figure 3B) showed obviously different viabilities on the solid medium with and without theophylline (Figures 3C and 3E). Then we mutated the catalytic core of TAP-1 and TAP-18 into TAP-1m and TAP-18m to inactivate their RNA-cleaving activity. Under the same reporting system and growth condition, TAP-1m and TAP-18m failed to grow on the plates with or without theophylline (see also Figure S2), which verified that *E. coli* harboring TAP-1 and TAP-18 survives through RNA self-cleavage after binding with theophylline. We also conducted FAM-labeled *trans*-cleaving experiments to verify that the *in vitro* cleavage efficiency of TAP-1 and TAP-18 were both promoted by theophylline (see also Figure S3). As shown in Figures 3D and 3F, TAP-1 demonstrated 1.2-fold and TAP-18 demonstrated 3.7-fold of promotion in *trans*-cleaving efficiency with 1 mM theophylline in the reaction buffer.

### Modulation Efficiency Quantification in Prokaryotic System

**Figure 4:**
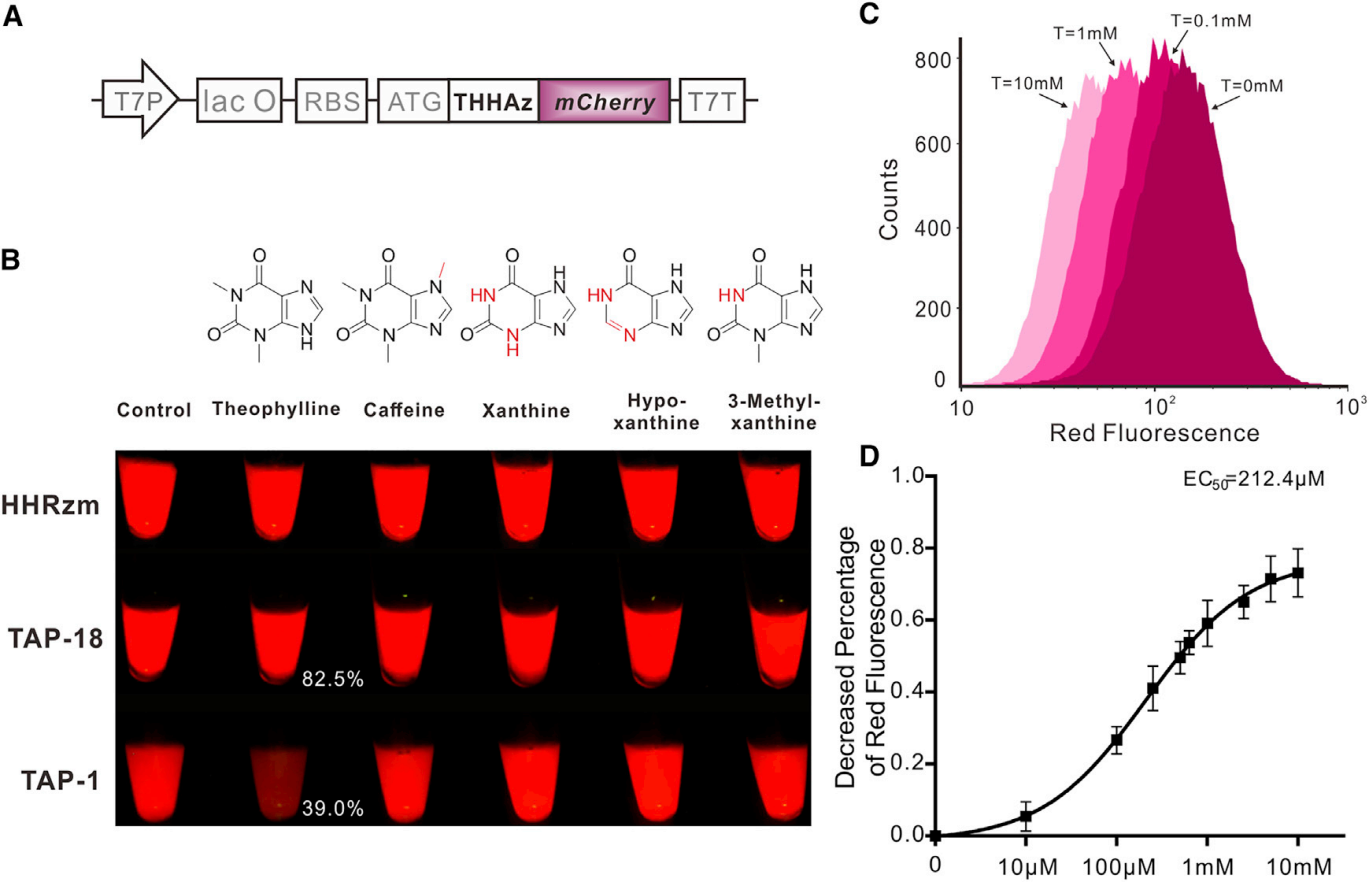
Modulation in Prokaryotic System (A) The strategy of HHAz fusion expression with mCherry in prokaryotic system. (B) The experiment to investigate the specificity of selected HHAzs. The molecular structure of each analog was illustrated above corresponding tubes. Red line and letters showed the difference from theophylline molecule. The remaining red fluorescence signal after theophylline addition was shown in the picture, and the control samples without adding molecules were set as 100%. (C) Cytometer histograms of red fluorescence signal produced by host cells harboring TAP-1 in reaction of 0 mM, 0.1 mM, 1 mM, and 10 mM theophylline, respectively from right to left. Quantitative data is supplemented in Figure S4C. (D) The decreased percentage of red fluorescence at different concentrations of theophylline were calculated. Decreased percentage of red fluorescence = RT^−^-RT^+^/RT^−^. RT^−^ = red fluorescent intensity without theophylline adding, and RT^+^ = the red fluorescent intensity with theophylline adding. The EC_50_ of TAP-1 is around 212.4 μM. All calculations were made using the GraphPad Prism software (GraphPad Software).

The IbsC-based cell survival experiment is hard to quantify intracellular downregulating efficiency of HHAz variants. Therefore, to obtain the accurate off-switching ability of HHAz with and without theophylline adding, the reporting protein IbsC was replaced with a low-background red fluorescent protein *mCherry* (Figure 4A). To verify the feasibility of this strategy, we first inserted HHRz and HHRzm at the 5′ end ORF of *mCherry*. As a result (see also Figure S4), the mRNA with and without self-cleaving ability caused a significant difference in red fluorescence signal, indicating that the self-cleavage of mRNA near 5′ end hinders the translation of *mCherry* and the fusion expression of HHRzm and *mCherry* didn’t compromise the red fluorescence signal. Then, TAP-1 and TAP-18 were individually inserted into the vector to fusion express with *mCherry*. As illustrated in Figure 4B, the fluorescent signals of *E. coli* cells containing TAP-1 and TAP-18 revealed significant downregulation in presence of 2 mM theophylline, which is visible to the naked eyes, while the same concentration of the analogs of theophylline such as caffeine, xanthine, hypoxanthine, and 3-methylxanthine cannot cause detectable downregulation of *mCherry*, indicating that both selected HHAz variants maintain the high specificity to theophylline. As TAP-1 afforded much better off-switching efficiency (61%) than TAP-18 (17.5%), we analyzed its responses to different concentrations of theophylline with flow cytometry. The intracellular HHAz TAP-1 was very sensitive to the exogenous regulator and responded significantly toward theophylline from 10 μM to 10 mM, causing the obvious downregulation of *mCherry* protein (see Figure 4C; Figure S4C). Based on the quantitative analysis, the EC_50_ (half-maximal effective concentration) of theophylline for HHAz variant TAP-1 is determined as 212.4 μM (Figure 4D). And the gene-regulation ability of TAP-1 could be greatly affected by introducing mismatches into the communication region of TAP-1(see Figure S5).

### Modulation Efficiency in Eukaryotic System

Considering the potential application in future gene therapy, since TAP-1 is obtained in *E. coli*, it is necessary to investigate their modulation ability in the eukaryotic system. Unlike in prokaryotic cells, the HHRz was inserted in the 5′ end of the mRNA of the targeted protein. In eukaryotic cells, HHRz was normally inserted in the 3′ end of mRNA, utilizing the self-cleavage of HHRz causing the loss of 3′ poly(A) tails, which destabilizes the mRNA, leading to the downregulation of *mCherry* protein (see Figure S6B). Thus, HHAz was individually inserted into the 3′ end of exogenous gene *mCherry*, and HEK293T cell was chosen to harbor the constructed vectors. As a result, TAP-1 showed much better gene-regulation results than TAP-18 (see Figure S6C) and maintained its specificity toward theophylline in the eukaryotic system (see Figure S6D).

**Figure 5:**
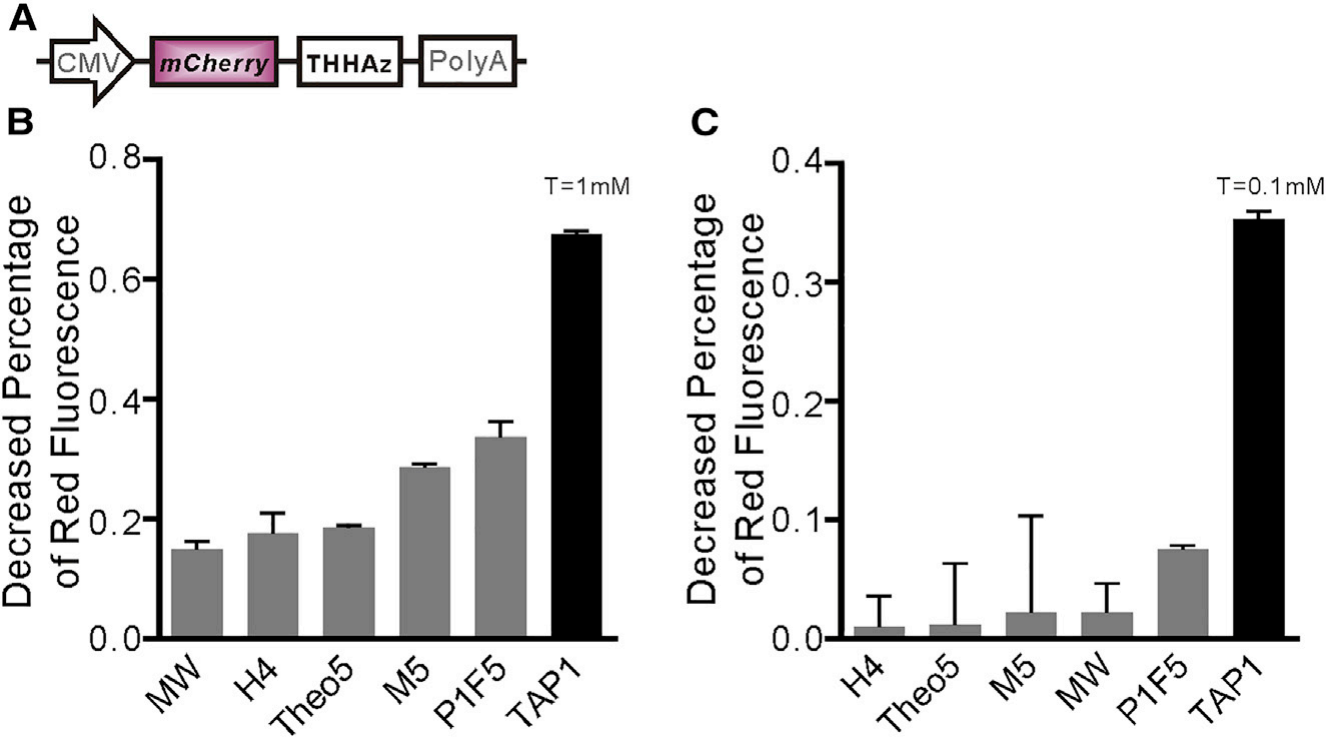
Modulation in Eukaryotic System (A) The strategy of theophylline-dependent HHAz downregulating mCherry expression in eukaryotic system. (B) Decreased percentage of red fluorescence in HEK293T with 1 mM theophylline. Decreased percentage of red fluorescence = RT^−^-RT^+^/RT^−^. RT^−^ = red fluorescent intensity without 1 mM theophylline adding, and RT^+^ = the red fluorescent intensity with 1 mM theophylline adding. (C) Decreased percentage of red fluorescence in HEK293T with 0.1 mM theophylline. Decreased percentage of red fluorescence = RT^−^-RT^+^/RT^−^. RT^−^ = red fluorescent intensity without 0.1 mM theophylline adding, and RT^+^ = the red fluorescent intensity with 0.1 mM theophylline adding.

In recent years, many theophylline-dependent HHAzs have been obtained and verified through different strategies, while no collective comparison has been conducted to figure out which one works in eukaryotic systems with more preferable efficiency. In the work conducted by Hartig et al.,^21^ the performance of theo-HHAz,^19^ P1F5,^21^ and 5.3^21^ were evaluated together, and P1F5 showed better performance. Similarly, theo-HHAz,^19^ L2bulgeOff2,^11^ and MW (not named in the original paper)^22^ were evaluated together by Pei et al.,^38^ in which MW showed better switching ability. Thus, to conduct a fair comparison and evaluate the performance of TAP-1 under a unified strategy, the chosen HHAz variants, M5,^20^ P1F5, MW, H4,^23^ and Theo5^17^ (shown in Figure 1C) have been inserted at the same position to downregulate the expression of *mCherry*, and HEK293T cell was chosen to harbor the constructed vectors. After transfection and incubation with 1 mM theophylline for 48 h,^17^^,^^21^^,^^25^ all variants downregulated red fluorescence signal effectively. M5 and Theo5, obtained through rational design, individually downregulated 28.6% and 18.7% of the red fluorescence signal. Moreover, MW, theo-H4, P1F5, and TAP-1, obtained through intracellular selection, individually downregulated 15%, 17.7%, 33.6%, and 67.5% of the red fluorescence signal (Figure 5B). Comparing with the quantitative data, the intracellular-selected HHAz variants demonstrated more positive downregulation efficiency than rational-designed ones, suggesting that intracellular selection is more reliable to obtain active HHAz variants to work in the cell.

To take the possible application in future gene therapy into consideration, a lower but safer concentration of theophylline, 0.1 mM, was set to compare the modulation performance of all HHAz candidates. As shown in Figure 5C, except TAP-1 and P1F5, the rest of the HHAz variants showed less than 3% of downregulation efficiency, which indicated that they barely react to the 0.1 mM of theophylline. Moreover, the downregulation efficiency of P1F5 reached 7.6%, which suggested its modest regulation ability toward 0.1 mM of theophylline. However, although the modulation efficiency decreased partly compared to 1 mM of theophylline, TAP-1 afforded a 35.3% decrease of the red fluorescence signal, maintaining its outstanding downregulation ability at 0.1 mM of theophylline. This result demonstrated that TAP-1 can regulate the target gene expression under a rather low concentration of theophylline as 0.1 mM in the eukaryotic system.

## Discussion

Without protein recruitment, artificial riboswitch realizes precise gene expression regulation through the specific binding between the RNA and exogenous small molecules, which is convenient for the regulations of transgene expression and the purposeful control of all RNA-related biotechnologies, such as microRNA-mediated RNA interference and CRISPR-mediated base editing. In the perspective of the application, hammerhead ribozyme-based theophylline-dependent aptazyme exhibits unique features. First, hammerhead ribozyme is a promising molecular tool in gene-regulation systems, due to its small size, typically less than 50 base pairs, which could be easily included in gene therapy vectors such as adeno-associated vectors (AVVs) with limited space for additional genes. Second, the theophylline aptamer exhibits particularly specific binding activity toward theophylline, instead of caffeine, which differs from theophylline by only one methyl group. Last but not least, theophylline is a common and safe ingredient in beverages such as tea, so it is possible in the future to modulate exogenous genetic therapy as easy as drinking a cup of tea. Besides, present commonly used methods, including viral vectors^39^ or non-viral methods,^40^ could be applied in the delivery of TAP-1 based exogenous DNA. As for the targeting of specific cells in gene therapy, tissue-specific promoters^41^ in the vector bearing TAP-1 or tissue-specific viruses^42^ can be used to transfer the TAP-1 based gene into the targeting cells.

For now, approaches to develop intracellular-applied hammerhead aptazyme are confined with rational design or FACS-based intracellular selection. Besides, the fluorescence-dependent phenotype of single host cells is easily affected by unintentional factors, which makes the selection process more easily influenced by false-positive signals. Here, we developed an intracellular selection strategy basing on a toxin protein *ibsC*, exhibiting several advantages as follows: (1) successfully converting the negative signal, i.e., downregulation of toxin *ibsC*, into a positive phenotype, i.e., colony growth on the solid plates; (2) selecting the HHAz variants with desired property through increasing selection pressure; (3) no dependence on sophisticated equipment. (4) realizing easy-operating and time- and labor-saving selection process. With these features, this proposed method is generally applicable to select HHR-based aptazymes to respond to different ligands, as well as all functional RNA molecules with self-cleaving ability.

With this strategy, a novel theophylline-dependent HHAz variants, TAP-1, have been obtained. After further evaluation of intracellular modulation efficiency with a red fluorescence protein reporting system, TAP-1 showed outstanding switching ability in both prokaryotic and eukaryotic systems with high specificity toward theophylline. Non-negligibly, among presently reported theophylline-dependent HHAz candidates, TAP-1 has been revealed to be equipped with the remarkable modulation range under the variable concentration of theophylline. Last but not least, TAP-1 can effectively regulate gene expression at a much lower concentration of theophylline, 0.1 mM, which is a safe concentration for the human body, indicating that TAP-1 would be a suitable candidate for clinic applications in gene therapy due to its ability to work at low concentration of theophylline. The work of using TAP-1 to regulate the release of therapeutic small interfering RNA (siRNA) molecules the assemble of CRISPR-Cas9-based genome-editing tools is currently underway in our lab.

## Materials and Methods

### Selection Procedure

All nucleotide acids are synthesized from Sangon Biotech (Shanghai, China). The DNA sequence of the randomized pool for CM of HHAz was as follows: 5′-ACGCAGGTACATCCAGCTGATGAGTCCCAAATAGGACGAAA(N2-5)ATACCAGCCGAAAGGCCCTTGGCAG(N2-5)TCCTGGATTCCACTGCG-3′. N2-5 is 2 to 5 randomized nucleotides in CM. 5′ end of HHRz was restriction enzymes site (BamHI); 3′ end of HHRz was restriction enzymes site (HindIII). The selection dsDNA pools were prepared from fusion PCR products of 3 fragments of DNA sequences. The first fragment is THL3-1, which is consistent. The second fragment, L3R3-6, bears the randomized sequences. The third fragment has three variants. THL3-2 is the third fragment for L3R3 and L3R6; similarly, THL3-3 is for L3R4 and THL3-4 is for L3R5. This combination is to avoid frameshift for IbsC (sequence, see Table S1). Fusion PCR was performed as follows: pre-denaturation at 95°C for 1 min, then 20 cycles (30 sec at 95°C, 30 sec at 53°C, 50 sec at 72°C of each cycle).

The selection vector was pT7Oi.^35^ DNA products were purified by AxyPrep PCR cleanup kit (Axygen) and double digested by BamHI and HindIII New England Biolabs (NEB) in 37°C for 3 h. Digested fragments of DNA pools were purified by AxyPrep PCR cleanup Kit (Axygen) again. DNA fragments of vector pT7Oi digested by the same restriction enzyme were extracted by AxyPrep DNA Gel Extraction Kit (Axygen). Digested products were ligated by NEB T4 ligase in 4°C overnight. The ligation products were extracted once with equal volume of phenol-chloroform extraction and then ethanol precipitated following recovery in 10 μL sterile deionized water. Electro transformed competent cells were prepared according to the manufacturer’s protocol (Bio-Rad) with moderate modifications. Purified ligation products were electro transformed into bacterial expression strain JM109(DE3) (Promega) and recovered in 37°C and 200 rpm for 1 h. All cells were harvested by centrifugation at 4,000 rpm for 5 min following coating on LB plate added with 100 μg/mL ampicillin (Sigma-Aldrich), 1 mM isopropyl β-D-1-thiogalactopyranoside (IPTG; Sigma-Aldrich), and 1 mM theophylline (Macklin, Shanghai, China). In the induction of IPTG, cells with potential active HHAz mutants would survive on the plate. By reapplying the survived colonies on the plates with and without theophylline at same time, it was testified that the different viability was caused by theophylline-modulated HHAz variants. After ensuring the difference viability between plates with and without theophylline adding, mutants were identified by DNA sequencing (Sangon Biotech).

### Quantification of Modulation Efficiency Based on Red Fluorescent Protein using FCM

The vector for red fluorescence quantification was the same structure as pT7Oi, only the toxin protein *ibsC* was replaced with red fluorescence protein *mCherry*. Thus, the quantification plasmid was named pT7Om. The recombinant vectors containing WT-HHRz, HHRzm, and selected variants were transformed into bacterial expression strain BL21(DE3) (TransGen, Beijing, China) and recovered in 37°C and 150 rpm for 1 h. 50 μL of transformed cells were coated on LB plates with ampicillin.

More than three monoclones of each structure were inoculated in 1.5 mL LB medium with ampicillin and cultivated in 37°C and 250 rpm until OD600 of culture medium reach 0.3. The cells in logarithmic phase were induced by IPTG (final concentration 1 mM) and divided into two portions. One is added with 1 mM final concentration theophylline; another is added same volume of sterilized water. They were both incubated in 37°C and 150 rpm for 18 h. Then *E. coli* were centrifuged at 4,000 rpm for 5 min and washed with PBS twice to abolish LB medium. Cell pellets were resuspended by PBS and analyzed by FCM (red fluorescence, excitation 561 nm, emission 610 nm). Since off-switching ability is measured on themselves with and without theophylline adding, it was assumed that the two portions of cells that coming from one colony had the similar conditions in their individual cells, as long as theophylline concentration wasn’t higher than 1 mM. Accidental errors were ruled out through repeated experiments.

The quantification plasmid used in eukaryotic cells was pmCherry-C1 (a gift from professor Hu Qinxue’s laboratory). 2.5 μg/well of the recombinant vectors containing WT-HHRz, HHRzm, and HHAz variants were parallelly transfected with Lipo2000 in Opti-MEM (OMEM, Thermo Fisher Scientific) into HEK293T cells (1.0 × 10^6^ /well) in the 6-well plates. After incubation for 4 h, medium was changed from OMEM into DMEM, supplemented with 10% FBS, penicillin (100 U/mL), streptomycin (100 mg/mL), and 0.1 mM or 1 mM theophylline. After incubation in 37°C and 5% CO_2_ for 48 h, cells were washed twice with PBS and collected for FCM analysis.

### Specificity Experiment

The recombinant vectors containing HHRzm, TAP-1, and TAP-18 were transformed into bacterial expression strain BL21(DE3) (TransGen) and recovered in 37°C and 150 rpm for 1 h. 50 μL of transformed cells were coated on LB plates with ampicillin. *E. coli* sample harboring the HHRzm, TAP-1, or TAP-18 came from the same colony individually. More than three monoclones of each structure were inoculated in 1.5 mL LB medium with ampicillin and cultivated in 37°C and 250 rpm until OD600 of culture medium reach 0.3. The cells in logarithmic phase were separated into 700 μL each induced by IPTG (final concentration 1 mM), theophylline (1 mM), xanthine (1 mM, Macklin), hypoxanthine (1 mM, Macklin), and 3-methylxanthine (1 mM, Macklin). Each sample was incubated in 37°C and 150 rpm for 18 h. After induction, the OD600 of each sample was tuned with blank LB medium to 1.5. 500 μL of *E. coli* from each sample was centrifuged to remove the LB medium and resuspended with 30 μL sterilized water. After the samples were prepared, red fluorescence signal was directly excited by a real-time blue light monitor (BD-BRV1, Ding Guo Prosperous, Beijing, China) with blue light of 485 nm and the picture was captured with a yellow filter in front of the camera.

As for the eukaryotic specificity experiments, 2.5 μg/well of the recombinant pmCherry-C1 vectors containing TAP-1 parallelly transfected with Lipo2000 in Opti-MEM (OMEM, Thermo Fisher Scientific) into HEK293T cells (2 × 10^5^ /well) in the 24-well plates. After incubation for 4 h, medium was changed from OMEM into DMEM, supplemented with 10% FBS, penicillin (100 U/mL), streptomycin (100 mg/mL), and 0.5 mM theophylline and its analogs individually. After incubation in 37°C and 5% CO_2_ for 24 h, cells were washed twice with PBS and collected for FCM analysis.

### Extracellular Experiments

As illustrated in Figure S3A, the self-cleaving HHAz variants are split into two parts at Loop1 and Loop2, and the interaction of Loop1 and Loop2 is replaced with totally complementary binding arms. The binding arms were 8 base pairs each, and S1 is a consistent strand. When the 5 end labeled E binds with S1, and the catalytic core is stabilized, then the cleaving is to be completed with magnesium ion adding. After cleaving, 5 end labeled E is shortened, and the mobility of E is changed, thus the uncleaved E is differentiable with cleaved E on the PAGE gel. Cleavage products were separated on 15% denaturing PAGE and analyzed by Typhoon FLA 7000 IP (GE Healthcare).

5′ FAM-labeled RNA sequences are synthesized from Tsingke (Beijing, China). Constant RNA strands S1 and S1m are *in vitro* transcribed from DNA double strands (synthesized in Sangon, Shanghai, China). DNA oligonucleotides containing the desired mRNA sequences were constructed to imbed T7 promoter sequence at 5′ end. A transcription reaction containing 0.5 mM of DNA template, 10 mM of NTP, T7 RNA polymerase buffer, and 1.25 U/μL T7 RNA polymerase (Thermo Fisher Scientific) were incubated at 37ºC for 6 h, followed by ethanol precipitation and purification by 10% PAGE. Sequences of templates were shown in Table S1.

The cleavage reaction of 1 nM FAM-labeled E sequence with 5 nM S1 was carried out in 50 mM Tris-HCl (pH 7.5) buffer containing 10 mM MgCl_2_ with or without 1 mM theophylline at 37°C. The cleavage reactions were stopped by the addition of 1 volume of stop buffer (80% [v/v] deionized formamide, 50 mM EDTA pH 8.0, 0.025% [w/v] bromophenol blue, 0.025% [w/v] xylene cyanole). Subsequently, the reactions were analyzed with 15% denaturing PAGE. The efficiency of the cleavage reactions was determined by quantitation of the grayscale in the substrates and product bands using a Typhoon Laser scanning imaging system via a phosphor imaging system. The gray gradient was analyzed by ImageQuant TL.

### Statistical Analysis

Results were expressed as mean ± SD. All calculations were made using the GraphPad Prism software (GraphPad software).

## Author Contributions

Conceptualization, Z.T.; Methodology, Z.T. and X.H.; Investigation, Q.P. and S.Z.; Formal Analysis, Q.P., S.Z., and Y.Y.; Writing – Original Draft, Q.P.; Writing – Review & Editing, Z.T.; Funding Acquisition, Z.T., X.H., and J.D.; Resources, X.C., F.D., Y.Y., and G.C.; Supervision, Z.T.

## Conflicts of Interest

The authors declare no competing interests.

## Supplemental Information

Document S1. Figures S1–S6 and Tables S1 and S2

Document S2. Article plus Supplemental Information
