# Supplementary material for: Intracellular Selection of Theophylline-Sensitive Hammerhead Aptazyme
Source: Mol Ther Nucleic Acids. 2020 Mar 13;20:400–8. doi: 10.1016/j.omtn.2020.03.001 (PMC7118274; doi:10.1016/j.omtn.2020.03.001)
Supplement: Document S1. Figures S1–S6 and Tables S1 and S2 [file mmc1.pdf]

OMTN, Volume *20*

Supplemental Information

Intracellular Selection of Theophylline-Sensitive Hammerhead Aptazyme

Qinlin Pu, Shan Zhou, Xin Huang, Yi Yuan, Feng Du, Juan Dong, Gangyi Chen, Xin Cui, and Zhuo Tang

Supplemental Information(SI)

Intracellular Selection of Theophylline-Sensitive Hammerhead Aptazyme

Qinlin Pu[1,2], Shan Zhou[1,2], Xin Huang[1], Yi Yuan[1], Feng Du[1], Juan Dong[1], Gangyi Chen[1], Xin Cui[1] and Zhuo Tang[1,*]

[1]Natural Products Research Center, Chengdu Institution of Biology, Chinese Academy of Science, Chengdu 610041, P.R. China.

[2]University of Chinese Academy of Sciences, Beijing 10049, P.R. China.

[*]Correspondence should be addressed to Zhuo Tang. (tangzhuo@cib.ac.cn). Fax: +86 28 8524 3250; Tel: +86 28 8524 3250

Contents

FigureS1-6

Table S1-2

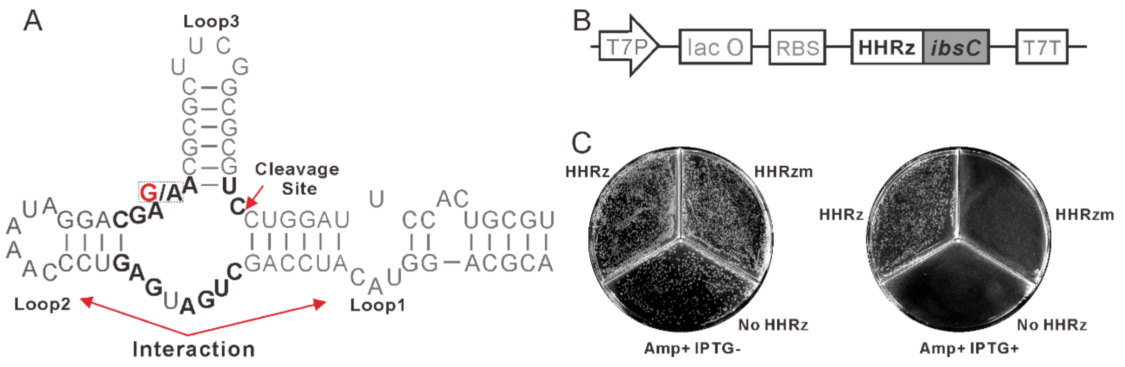

Figure S1:

(A) The illustration of HHRz from *Schistosoma Mansomi*. The black bold letters are the conventional catalytic core of HHRz. HHRz is inactivated when the boxed A mutated into G. Single red arrow shows the self-cleaving site of HHRz. The double red arrow shows the interaction between Loop1 and Loop2, which is the insurance of high self-cleavage efficiency.

(B) The strategy of HHRz fusion expressing with ibsC.

(C) The result of different viability for E.coli transferred with HHR, HHRzm and no HHRz plasmids. Only E.coli transferred with HHRz inserted plasmid survived on the plate with IPTG. The concentration of ampicillin is 100 μg/ml, and IPTG is 1 mM.

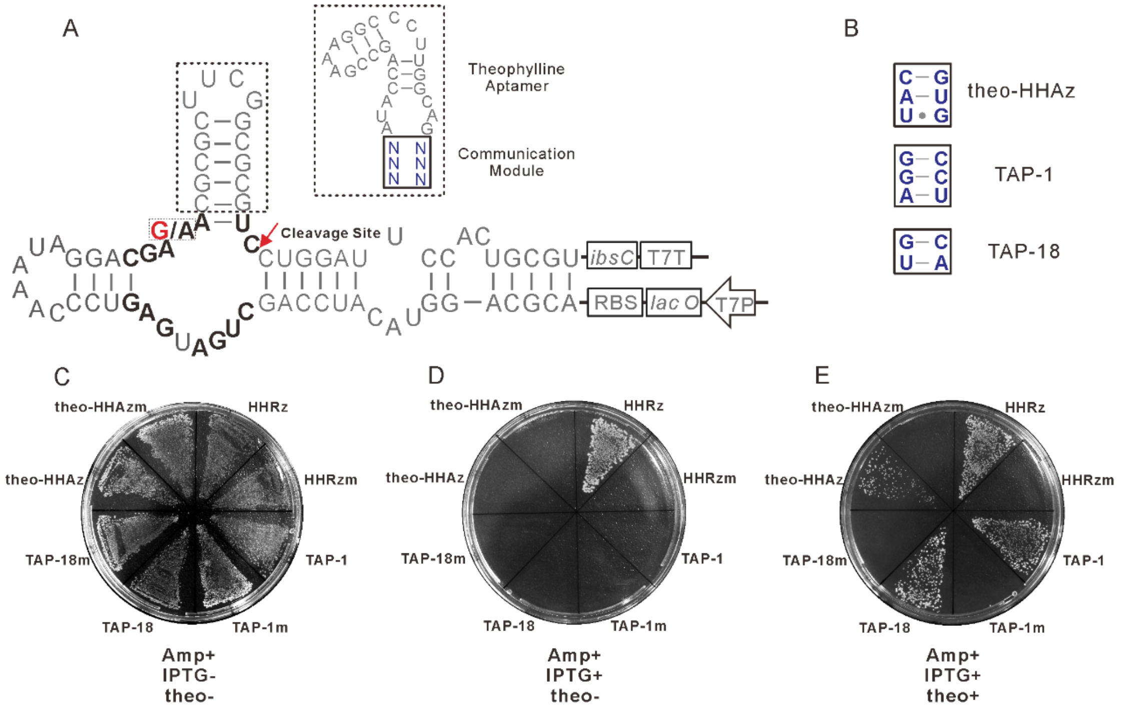

Figure S2:

(A) The strategy of inserting HHRz, HHRzm, TAP-1, TAP-1m, TAP-18, TAP-18m, theo-HHAz, theo-HHAzm individually to fusion express with ibsC. When boxed A14 in catalytic core mutated into G14 in red, the self-cleaving active structures are inactivated.

(B) The illustration of CM domain of TAP-1, TAP-18 and theo-HHAz, and the rest of the nucleotides are the same as illustrated in A. C, D, E. 8 variants were inserted at the same site in the vector, and transferred into JM109DE3 individually at the same time. After recovering in 37 ºC for 1 h, same amount of E.coli was applied on the according area.

(C) The solid medium was added with 100 μg/ml ampicillin.

(D) The solid medium was added with 100 μg/ml ampicillin, 1 mM IPTG.

(E) The solid medium was added with 100 μg/ml ampicillin, 1 mM IPTG and 1 mM theophylline. The comparison between D and E showed that the self-cleavage efficiency was promoted after theophylline adding. When the self-cleaving ability was mutated, no bacterial colony was identified on the plate, referring that the cell survival is dependent on the self-cleavage ability.

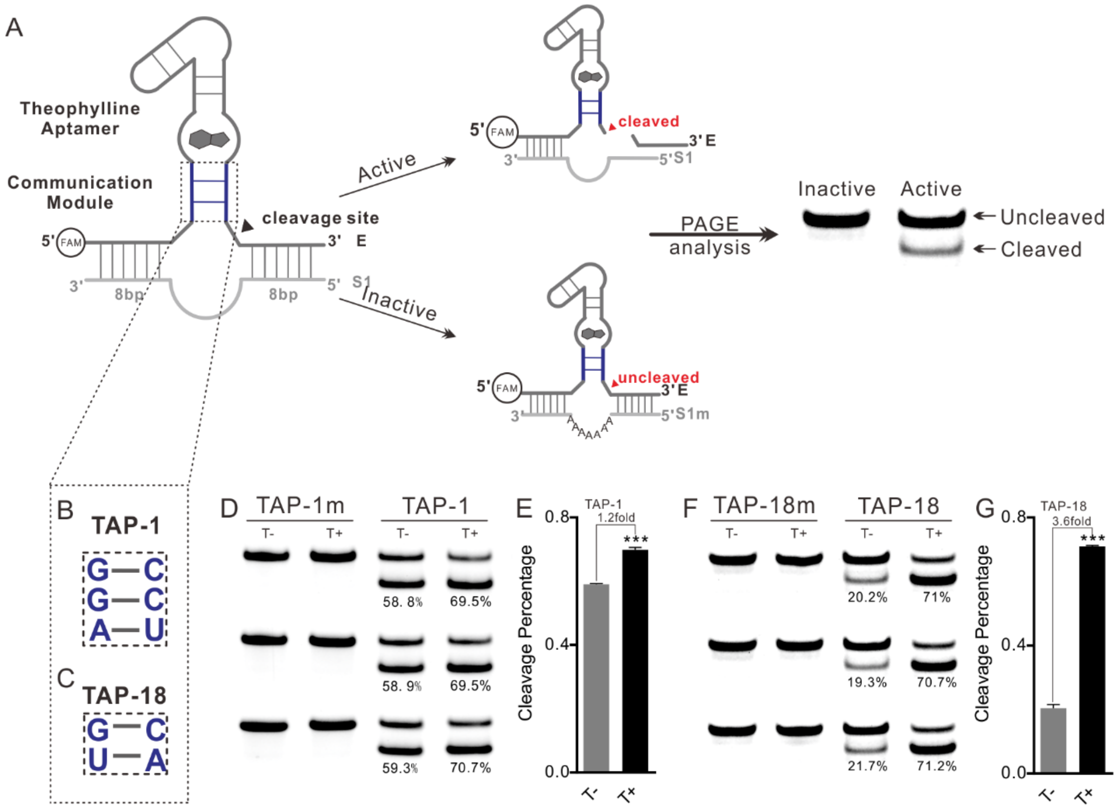

Figure S3:

(A) The illustration of the extracellular cleaving experiments.

(B) The illustration of CM domain of TAP-1, which is consistent with the self-cleaving HHAz variant utilized intracellularly.

(C) The illustration of CM domain of TAP-18, which is consistent with the self-cleaving HHAz variant utilized intracellularly.

(D) Three repeated cleavage reaction of TAP-1 and TAP-1m with or without theophylline adding. The fraction of cleaved TAP-1 were quantified and calculated with ImageQuant TL.

(E) The bar chart of cleavage percentage of TAP-1 with and without theophylline. The cleavage was significantly promoted after theophylline adding. p<0.001,***.

(F) Three repeated cleavage reaction of TAP-18 and TAP-18m with or without theophylline adding. The fraction of cleaved TAP-18 were quantified and calculated with ImageQuant TL.

(G) The bar chart of cleavage percentage of TAP-18 with and without theophylline. The cleavage was significantly promoted after theophylline adding. p<0.001,***.

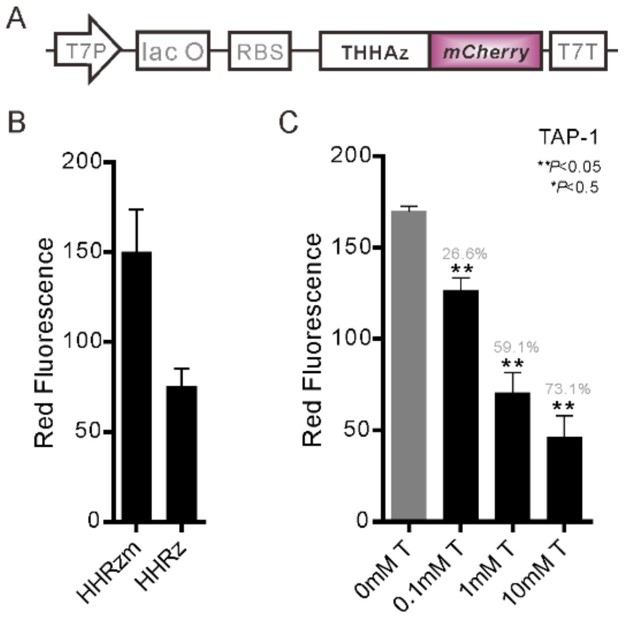

Figure S4:

(A) The strategy of quantifying modulation efficiency of HHAz variants in prokaryotic system. HHRz, HHRzm and HHAz variants are inserted to fusion expression with mCherry. Vectors bearing individual variants were transferred into BL21DE3. After a single colony with individual variants was incubated in LB to reach 0.3 of OD600, they were equally divided into two parts. One of them was added with 1mM of final concentration of IPTG, and another was added with the same amount of IPTG plus 1 mM or 0.1 mM of final concentration of theophylline. Then, both of them were incubated in 37 ºC for 18 h at 150 rpm, and then individual red fluorescence intensity was analyzed through FCM.

(B) The red fluorescence intensity of cells expressing HHRz and HHRzm, verifying the feasibility of the quantifying strategy.

(C) The red fluorescence of cells harboring TAP-1. Three groups of cells were collected at the same time. **, p<0.05; *, p<0.5. All calculations were made using the GraphPad Prism software (GraphPad software, Inc.).

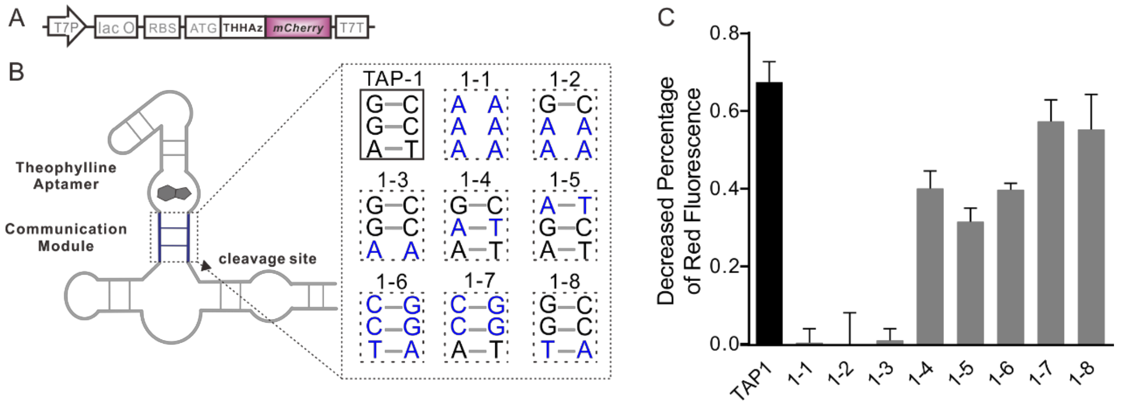

Figure S5:

(A)The construction of modulation strategy in E.coli.

(B)The illustration of designed variants.

(C)The bar chart of modulation percentage of each variants at 1mM of theophylline. Decreased percentage of red fluorescence=RT^{-}-RT^{+}/RT^{-}.

As shown in Figure S5, unmatched base pairs replaced the communication region as 1-1. As expected, 1-1 lost its regulation ability, which testified that the sequence in the communication region formed binding as predicted. Nevertheless, the modulation ability didn't recover as the matched base pairs were recovered one by one as 1-2 and 1-3, which verified that the stability in the communication region is critical for TAP-1. Next, we replaced the G-C pairs individually with slightly weaker binding base pair A-T to improve the flexibility of the communication module for better modulation ability. It turned out that neither variant 1-4 or 1-5 realized improved modulation ability, which indicated that the improved flexibility doesn't help with the modulation ability of TAP-1. Thirdly, as the stability and the flexibility of TAP-1 stayed the same, we examined the sequence specificity by transverse base pairs in the communication module, turning TAP-1 into 1-6, 1-7 and 1-8. Consequently, the regulation ability of the variants is all weakened compared to TAP-1, which verified the sequence specificity of TAP-1 in the communication region. Conclusively, by introducing various mutations into the communication region of TAP-1, it is verified that the specified sequence and stable binding in the communication module ensured its regulation ability.

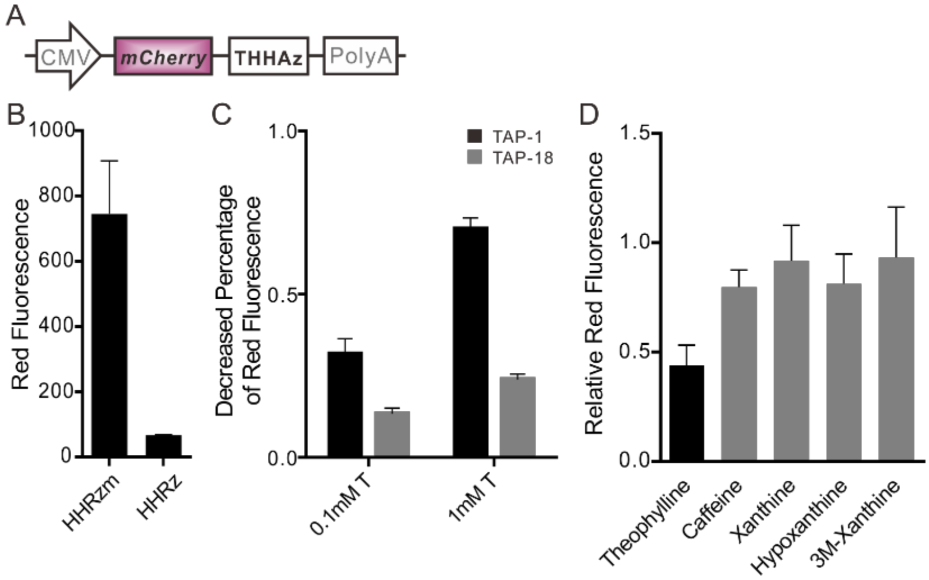

Figure S6:

(A) The strategy of quantifying modulation efficiency of HHAz variants in eukaryotic system. HHRz, HHRzm and HHAz variants are inserted at the 3'end of mCherry.

(B) The red fluorescence intensity of cells expressing HHRz and HHRzm, verifying the feasibility of the quantifying strategy.

(C) The decreased percentage of red fluorescence of TAP-1 and TAP-18 at 0.1mM and 1mM of theophylline. Decreased percentage of red fluorescence=RT^{-}-RT^{+}/RT^{-}.

(D) The relative red fluorescence of TAP-1 in reaction of Theophylline and its analogues. Relative Red Fluorescence=Red Fluorescence with small molecular adding/Red Fluorescence without small molecular adding. Drastically decreased red fluorescence was only observed in the group that was added with theophylline, while the rest groups added with the same concentration of analogues only had slight fluctuations.

Name	Sequences
HHR-F	GCTGGATCCCAGGTACATCCAGCTGATGAGTCCCAAATAGGACGAAACGCGCTTCGG
HHR-R	CGAAAGCTTCGCAGTGGAATCCAGGACGCACCGAAGCGCGTTTCGTCC
HHRm-F	GCTGGATCCCAGGTACATCCAGCTGATGAGTCCCAAATAGGACGAGACGCGCTTCGG
THL3-1	GCTGGATCCACGCAGGTACATCCAGCTGATGAGTCCCAAATAGGACGAAA
L3R3-2	AATAGGACGAAANVATACCAGCCGAAAGGCCCTTGGCAGNNTCCTGGATTCCA
L3R4-2	AATAGGACGAAANVNATACCAGCCGAAAGGCCCTTGGCAGVNNTCCTGGATTCCA
L3R5-2	AATAGGACGAAANVNNATACCAGCCGAAAGGCCCTTGGCAGNNVNTCCTGGATTCCA
L2R6-2	AATAGGACGAAANVNNVATACCAGCCGAAAGGCCCTTGGCAGNVNNNTCCTGGATTCCA
THL3/2-3	CGAAAGCTTGCAGTGGAATCCAGGA
THL3/3-3	CGAAAGCTTCGCAGTGGAATCCAGGA
THL3/4-3	CGAAAGCTTACGCAGTGGAATCCAGGA
theo-HHAz-2	AATAGGACGAAATACATACCAGCCGAAAGGCCCTTGGCAGGTGTCCTGGATTCCA
TAP-1-2	AATAGGACGAAAAGGATACCAGCCGAAAGGCCCTTGGCAGCCTTCCTGGATTCCA
TAP-18-2	AATAGGACGAAATGATACCAGCCGAAAGGCCCTTGGCAGCATCCTGGATTCCA
P1-F5-2	AATAGGACGAAAGCCATACCAGCCGAAAGGCCCTTGGCAGGGTTCCTGGATTCCA
5.3-2	AATAGGACGAAAAATCATACCAGCCGAAAGGCCCTTGGCAGGATTTCCTGGATTCCA
MW-2	AATAGGACGAAACCAGCATACCAGCCGAAAGGCCCTTGGCAGGTTGATCCTGGATTCCA
L2bulgeOff1-1	GCTGGATCCCGAGCTGTCACCGGATG
L2bulgeOff1-2	TGTCACCGGATGTGCTTTCCGGTCTGATGAGTCCGTGTTGCTGATACCAGCATCGTCTG
L2bulgeOff1-3	CGAAAGCTTGAGCTGTTTCGTCCTCGTCCACTGCTGCCAAGGGCATACAGACGATGCTG
M5-2	AATAGGACGAAACACATACCAGCCGAAAGGCCCTTGGCAGTTGTCCTGGATTCCA
3way theoHHAz-1	GCTGGATCCCAGGTACTAGCAGCTGATGAGTCCCAAATAG
3way theoHHAz-2	ATGAGTCCCAAATAGGACGAAACGCCGAATGGCGTCCTGTTCCGATACCAGCCG
3way theoHHAz-3	CGAAAGCTTCCCAGTGGATAGATACTCCGCTGCCAAGGGCCTTTCGGCTGGTATCGGAA
theoH4-2	AATAGGACGAAATGAAAATACCAGCCGAAAGGCCCTTGGCAGTTTAATCCTGGATTCCA
Theo5-2	AATAGGACGAAAGGAATACCAGCCGAAAGGCCCTTGGCAGTCTTCCTGGATTCCA
E-TAP-1	GUAGGGACGAAAAGGAUACCAGCCGAAAGGCCCUUGGCAGCCUUCCUGGAUCC
E-TAP-18	GUAGGGACGAAAUGAUACCAGCCGAAAGGCCCUUGGCAGCAUCCUGGAUCC
S1	CGAAATTAATACGACTCACTATAGGGATCCAGCTGATGAGTCCCTACATC
S1m	CGAAATTAATACGACTCACTATAGGGATCCAGAAAAAAAGTCCCTACATC

Table S1:

The synthesized sequences are listed in this table. The complete sequence of HHRz is obtained by the extension of HHR-F and HHR-R. The complete sequence of HHAz variants are synthesized by fusion PCR of 3 pieces. The THL3-1 is the constant sequence. According to the different length of HHAz variants, the choice of THL3-3 is different. The key point is to combine the 3 pieces of fragments into the multiple of 3 to avoid the frame shift of protein expression.

The selection library is made of 256(2bp), 2304(3bp), 49152(4bp) and 589824(5bp) variants, which is totally no more than 10^6 variants (the randomized area was boxed in the figure below). The selection plasmids were constructed by inserting the corresponding synthesized DNA fragments that contain the randomized communication modules. The corresponding plasmid library was transformed into E. coli cells to construct about 10^7 bacteria library, which covered all variants theoretically.

Name	Sequences	Repitition
theo-HHAz	ACGCAGGTACATCCAGCTGATGAGTCCCAAATAGGACGAAATACATACCAGCCGAAAGGCCCTTGGCAGGTGTCCTGGATTCCACTGCGT	2
TAP-1	--AGG---------------------------CCT-------------------	1
TAP-2	--TAT---------------------------ATA-------------------	3
TAP-3	--TG---------------------------CT-------------------	2
TAP-4	--TA---------------------------AT-------------------	2
TAP-5	--TG---------------------------TT-------------------	1
TAP-6	--AA---------------------------TT-------------------	1
TAP-7	--AACTA---------------------------CGGTC-------------------	1
TAP-8	--TGGTC---------------------------CCTCC-------------------	1
TAP-9	--TG---------------------------AT-------------------	1
TAP-10	--GGA---------------------------CAT-------------------	1
TAP-11	--GC---------------------------GC-------------------	1
TAP-12	--TCCCC---------------------------TAGTC-------------------	1
TAP-13	--GGA---------------------------ATT-------------------	1
TAP-14	--TG---------------------------GT-------------------	1
TAP-15	--TGA---------------------------AGC-------------------	1
TAP-16	--TGGT---------------------------CCAT-------------------	1
TAP-17	--CACT---------------------------AGCG-------------------	1
TAP-18	--TG---------------------------CA-------------------	1
TAP-19	--TAT---------------------------AAT-------------------	1
TAP-20	--TAGCC---------------------------CCACC-------------------	1
TAP-21	--GA---------------------------AA-------------------	1
TAP-22	--TC---------------------------AT-------------------	1
TAP-23	--TCG---------------------------CTT-------------------	1
TAP-24	--AGC---------------------------GAT-------------------	1
TAP-25	--TA---------------------------GC-------------------	1
TAP-26	--CCGC---------------------------TAAC-------------------	1
TAP-27	--GC---------------------------CC-------------------	1
TAP-28	--CCGGG---------------------------TCCTA-------------------	1
TAP-29	--TCG---------------------------GCA-------------------	1
TAP-30	--GAGTC---------------------------AGTCG-------------------	1
TAP-31	--TG---------------------------CC-------------------	1
TAP-32	--TAG---------------------------AAT-------------------	1
TAP-33	--ACTCC---------------------------TAGCT-------------------	1
TAP-34	--GC---------------------------CA-------------------	1
TAP-35	--TC---------------------------TT-------------------	1
TAP-36	--GC---------------------------AC-------------------	1
TAP-37	--ACCG---------------------------TAGA-------------------	1
TAP-38	--TG---------------------------AT-------------------	1

Table S2:

The selected sequences are listed in table S2. Theo-HHAz is given the complete sequence as an example. Illustrated in the table are communication module sequences of the variants, and identical sequences in the variants were replaced with dashes. The communication module sequence of theo-HHAz, TAP-1 and TAP-18 were shown in red. Repetition represents the frequency that the variant was identified.
