# Supplementary material for: Intracellular Selection of Theophylline-Sensitive Hammerhead Aptazyme
Source: Mol Ther Nucleic Acids. 2020 Mar 13;20:400–8. doi: 10.1016/j.omtn.2020.03.001 (PMC7118274; doi:10.1016/j.omtn.2020.03.001)
Supplement: Document S2. Article plus Supplemental Information [file mmc2.pdf]

Molecular Therapy
Nucleic Acids
Original Article

Intracellular Selection of Theophylline-Sensitive Hammerhead Aptazyme

Qinlin Pu,[1,2] Shan Zhou,[1,2] Xin Huang,[1] Yi Yuan,[1] Feng Du,[1] Juan Dong,[1] Gangyi Chen,[1] Xin Cui,[1] and Zhuo Tang[1]

[1]Natural Products Research Center, Chengdu Institution of Biology, Chinese Academy of Science, Chengdu 610041, P.R. China; [2]University of Chinese Academy of Sciences, Beijing 10049, P.R. China

Hammerhead ribozyme-based aptazyme (HHAz), inheriting the advantages of small size and high efficiency from the RNA-cleaving ribozyme and the specific recognition ability of aptamers to specific targets, exhibits the huge potential to be a transgene expression regulator. Herein, we report a selection strategy for HHAz by using a toxin protein IbsC as the reporter to offer a positive phenotype, thus realizing an easy-operating, time- and labor-saving selection of HHAz variants with desired properties. Based on this strategy, we obtained a new HHAz (TAP-1), which could react sensitively toward the extracellular regulatory molecule, theophylline, both in prokaryotic and eukaryotic systems. With fluorescent protein reporter, the intracellular switching efficiencies of TAP-1 and other reported theophylline-dependent HHAzs has been quantitatively evaluated, showing that TAP-1 not only exhibits the best downregulating ability at high concentration of theophylline but also maintains high activity with 0.1 mM theophylline, which is a safe concentration in the human body.

INTRODUCTION

Riboswitches, first termed in 2002,[1] are *cis*-acting RNA structures that modulate gene expression upon specific ligand binding without protein engagement. Natural riboswitches, though they commonly exist *in vivo*, are confined to bind with intracellular molecules to regulate metabolic-related gene expression, whereas purposeful gene expression control with exogenous ligands is largely dependent on the development of artificial riboswitches.[2] It is well accepted that riboswitches are composed of an aptamer domain and an expression platform. The aptamer domain is for specific recognizing of ligands; the expression platform is for converting the aptamer-ligand recognition into the regulation of the gene expression.[3] Riboswitches regulate gene expression commonly by either allosteric or catalytic principles.[4] RNA-cleaving ribozymes are often utilized to construct artificial riboswitches. Among diverse candidates, hammerhead ribozyme (HHRz, see Figure 1A) stands out for its advantages of small size, simple structure, and high modularity, and it has already been utilized as a safe and highly efficient molecular tool for knocking down of gene expression.[5] Moreover, HHRz-based riboswitch is also termed as hammerhead aptazyme (HHAz), which is HHRz appended with an aptamer domain to modulate its self-cleavage.[6] Owing to the development of the systematic evolution of ligands by exponential enrichment (SELEX),[7] RNA aptamers are massively described, which makes it theoretically possible to develop a specific RNA aptamer for any exogenous ligand. For now, only a few RNA aptamers obtained through SELEX have been verified to maintain their binding activity intracellularly, such as tetracycline aptamer,[8] neomycin aptamer,[9] tobramycin aptamer,[10] and theophylline aptamer.[11] On account of the possibility of future applications in gene therapy, gene expression regulation with aminoglycoside antibiotic ligands, such as tetracycline and neomycin, is possible to pose potential threats of antibiotic abuse toward public health. But, theophylline, a common medicine for the treatment of chronic asthma, is a safer ligand to use. Besides, a safe amount of theophylline is easily accessible in common beverages, such as tea.

The binding of certain ligands either promotes or hinders the self-cleavage of HHAzs. The promotion of self-cleavage is mainly realized by the formation of the stable catalytic core of HHRz,[12] while various mechanisms could be utilized to hinder the self-cleavage.[13] Among these HHAz variants, the aptamer domain is generally appended to HHRz through a communication module (CM; see Figure 1B). A CM is expected to be a proper transmitter that transmits the ligand-binding signal to the catalytic core of HHRz through a conformational change.[14] A CM that is too flexible will trigger no catalytic cleavage even with a ligand binding. On the contrary, a CM that is too stable will cause self-cleavage without a ligand binding. Two different methods, rational design and intracellular selection, are employed to obtain variants with diverse CMs. The first artificial HHAz is constructed through rational design, and verified *in vitro* by Breaker et al.,[15] while they only work extracellularly. Notably, many theophylline-dependent HHAz variants obtained through calculation-assisted rational design, are well functioned as regulation elements during the gene expression process in eukaryotic systems.[16,17] However, the intracellular environment is so complicated

Received 11 February 2020; accepted 10 March 2020;
https://doi.org/10.1016/j.omtn.2020.03.001.

Correspondence: Zhuo Tang, Natural Products Research Center, Chengdu Institution of Biology, Chinese Academy of Science, Chengdu 610041, P.R. China.
E-mail: tangzhuo@cib.ac.cn

 © 2020 The Author(s).
This is an open access article under the CC BY license (http://creativecommons.org/licenses/by/4.0/).

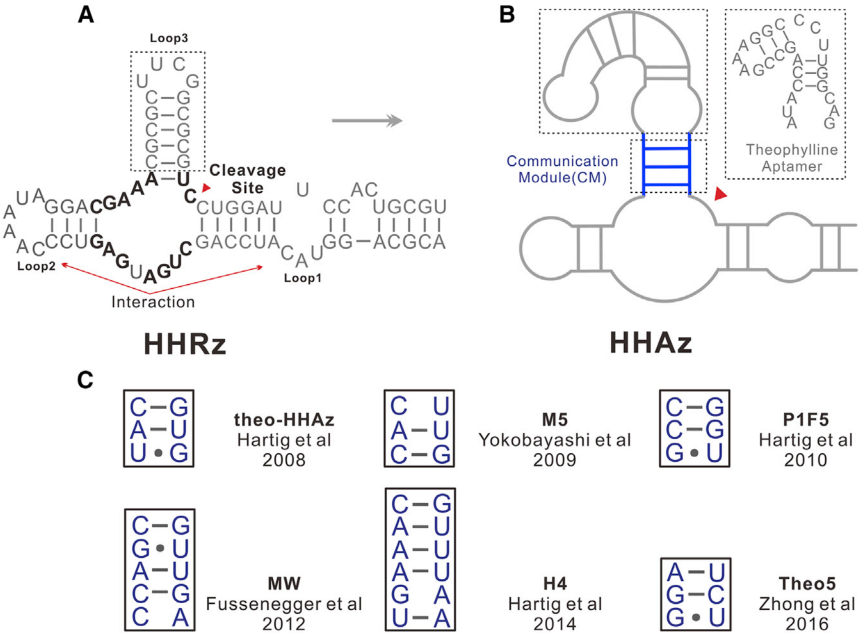

Figure 1. Illustration of Secondary Structures

(A) The secondary structure of a hammerhead ribozyme from *Schistosoma Mansoni*. (B) The illustration of a theophylline-dependent hammerhead aptazyme. (C) A list of communication modules of reported theophylline-dependent HHAzs that have been mentioned for modulation ability comparison in this report.

that it is hard to predict with theoretical thermodynamic parameters. On the contrary, the intracellular selection is more suitable for directly obtaining HHAz variants that target intracellular applications. The strategy of intracellular selection is often engaging with fluorescence protein such as *eGFP* or *mCherry* as its reporter. What's more, fluorescence-activated cell sorting (FACS) is utilized frequently among these intracellular selection strategies.[13,18] During the selection process, host cell with a decreased or increased intensity of fluorescence signal, caused by self-cleavage of an aptazyme after ligand binding, is often selected with FACS. However, the fluorescence signal of single host cell is easily affected by unintentional factors. Besides, FACS-based intracellular selection is highly dependent on the equipment of high-speed cell sorting flow cytometer (FCM). Moreover, with the increasing selection library, the selection procedure becomes increasingly time and labor consuming, adding much inconvenience to the selection procedure. Therefore, a simple and easy-operating intracellular selection strategy is highly desirable.

In the recent two decades, many theophylline-dependent HHAz variants were developed through rational design and intracellular selection strategies[17,19–23] (see Figure 1C), and these variants also achieved regulation capability in many fields. To exemplify, in 2008, theophylline-dependent HHAz was utilized to construct high-order cellular devices for information processing;[24] in 2009, the process of RNA interference was realized to be conditionally controlled by theophylline-dependent HHAz variants;[20] in 2010, it was realized to regulate the proliferation of mammalian T cell by using theophylline-dependent HHAz;[25] in 2012, theophylline-dependent HHAz was applied to *in vivo* screening of caffeine demethylase enzyme;[26] in 2017, the effectiveness of nuclease Cas9 was realized to be modulated by theophylline-dependent HHAz for off-targeting control;[27] last but not least, in gene therapy, adenovirus-based gene expression and safety control is also involving the modulation of theophylline-dependent HHAz variants.[28–30] When it comes to applying a certain theophylline-dependent HHAz variant, which one to choose becomes a question because no proper comparison is conducted. Besides, we identified a problem that the experimental concentration of theophylline is as high as 2–5 mM to realize certain regulation effects. However, as reported in clinical researches,[31] more than 20 mg/L (around 0.11 mM) of theophylline in blood will cause many adverse effects of anorexia, nausea, and vomiting, sometimes seizure in adults. Therefore, to take the further application of theophylline-dependent-HHAz-based gene therapy into consideration, it is urgent to screen HHAz variants that react sensitively under the safe concentration of theophylline.

Herein, we report a selection strategy for HHAz by using a toxin protein IbsC as the reporter to offer a positive phenotype, which makes the selection of desired HHAz conveniently and efficiently. Based on this strategy, we obtained a new theophylline-dependent HHAz variant, which could react more sensitively toward theophylline with a wider concentrate range than the reported theophylline-dependent HHAz variants in both prokaryotic and eukaryotic systems.

RESULTS

Selection Strategy Based on a Toxin Protein IbsC

In our intracellular selection strategy, *Escherichia coli* (*E. coli*) was chosen to harbor the randomized pool for intracellular selection, because every *E. coli* can only be transformed by one plasmid. Since the self-cleaving efficiency determines the phonotype of each host cell, the higher the self-cleaving efficiency, the less the corresponding protein expression, which makes it a negative signal for the given phenotype. The positive phenotype is more desirable than a negative signal since undesirable factors easily cause negative signals. IbsC[32] is an *E. coli*-originated toxin protein. It is a highly hydrophobic peptide, which anchors in the inner membrane of the bacterium after expression.[33] The overexpressed IbsC stagnates the growth status of *E. coli* by depolarizing the cell membrane and compromising the integrity of the cell envelope.[34] Moreover, extensive mutations are highly tolerable for IbsC, and it is feasible to fusion express with other protein at its N terminus without losing its toxicity. For instance, IbsC was reported to fusion express with wild-type HHRz (HHRz) and inactivated HHR (HHRzm, harboring an A14 to G14 in the catalytic core,

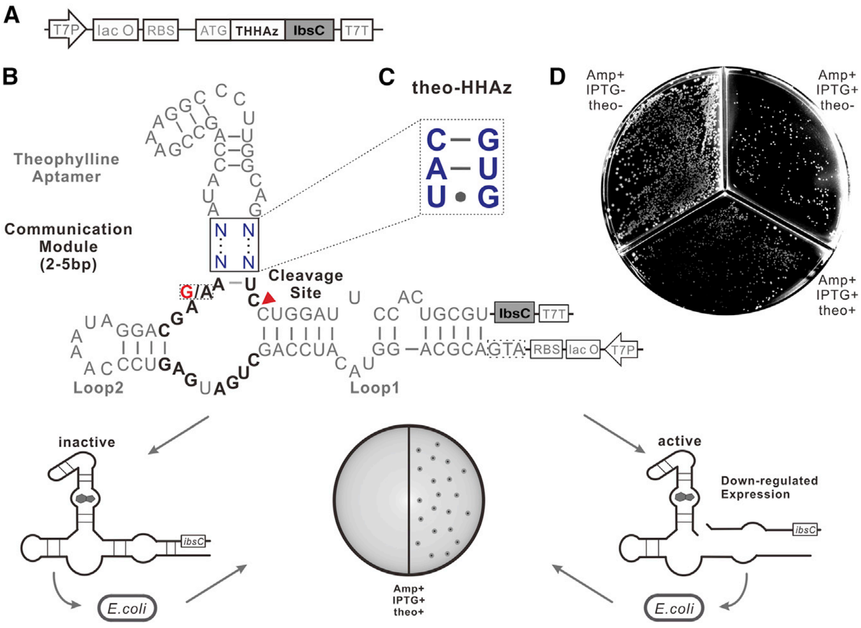

Figure 2. Selection Strategy

(A) The illustration for construction of selection vector basing on toxin protein IbsC. The HHAz randomized library is designed to fusion express with IbsC at its N terminus. (B) The illustration of the selection strategy. The boxed blue N is the randomized CM domain. 2–5 base pairs of randomized sequence were designed into the CM domain for self-cleaving modulation. The selection plate was added with 100 μg/mL ampicillin, 1 mM IPTG, and 0.5 mM theophylline. (C) The illustration of CM domain of theo-HHAz. (D) The result of different viability for *E. coli* transferred with theo-HHAz inserted plasmid on the solid medium added with and without theophylline. The concentration of ampicillin is 100 μg/mL, IPTG is 1 mM, and theophylline is 1 mM.

see Figure 2B) at the N terminus, which doesn't compromise its toxicity[35] (see also Figure S1). With these characteristics, the toxin protein IbsC is qualified for converting the negative signal, i.e., downregulation of IbsC, into a positive signal, i.e., colony growth on the solid plates.

As shown in Figure 2A, between the ribosomal binding site (RBS) and the open reading frame (ORF) of IbsC, the randomized pool of HHAz variants is inserted to fusion express with the toxin protein IbsC at its N terminus. They are both under the control of lacO operator. Besides, it has been reported that the interaction between Loop1 and Loop2 is the insurance of highly efficient self-cleavage for HHRz.[36,37] Therefore, the theophylline aptamer is designed to replace Loop3, and link to the catalytic core of HHRz through a randomized CM, leaving the Loop1 and Loop2 interaction undisturbed (see Figure 2B). When the expression is induced, as long as an invalid HHAz structure in the random pool fails to cleave itself, the downstream toxin protein IbsC will be expressed. Consequently, no visible colony expressing the invalid structure will grow on the Petri dish added with isopropyl β-D-1-thiogalactopyranoside (IPTG) and theophylline. On the contrary, as long as an HHAz structure in the random pool cleaves itself efficiently upon binding theophylline, the toxin protein IbsC will fail to express, resulting in the survival of corresponding bacteria colony on the Petri dish added with IPTG and theophylline. After sequencing the transferred vector from the very harvested bacteria, the sequence of the efficient HHAz variant can be obtained.

Before selection, it is necessary to verify the feasibility of the strategy that theophylline can regulate the expression of IbsC, resulting in different survival rates between the solid medium plates added with and without theophylline. Therefore, theo-HHAz (Figure 2C), reported by Hartig et al.[19] in 2008, was inserted to testify whether theophylline improves the self-cleavage efficiency of HHAz, downregulating the toxin protein IbsC. As shown in Figure 2D, *E. coli* transferred with theo-HHAz inserted plasmids exhibited different viability between the solid medium plates added with and without theophylline. This result verified the feasibility of our strategy.

Intracellular Selection Based on Toxin Protein IbsC

Based on the toxic protein IbsC, we started a systematic investigation. 2–5 base pairs of randomized CM domain were constructed into the recombinant plasmids and transferred into *E. coli* (designed randomized sequences were displayed in Table S1). Then, the library was incubated on the solid Luria-Burtani (LB) plate with 100 μg/mL ampicillin, 1 mM IPTG, and theophylline. With the induction of IPTG, the fusion mRNA of HHAz variants and *ibsC* would be massively transcribed. Only when the ones efficiently cleave themselves after binding theophylline will they survive on the plates and grow into a visible colony. From the 10^7 library, we harvested loads of colonies that have helped *E. coli* survive on the plate with 2 mM theophylline. To increase the selection pressure, we mixed the survived colonies again and then incubated them on the plates with 1 mM theophylline. The same procedure was repeated on the plates with 0.5 mM, 0.2 mM, and 0.1 mM in turn. In the end, we obtained 38 HHAz variants from the selection procedures (see Table S2). After reapplying the selected variants on the plates with and without theophylline at the same time, TAP-1 (Figure 3A) and TAP-18 (Figure 3B) showed obviously different viabilities on the solid medium with and without theophylline (Figures 3C and 3E). Then we mutated the catalytic core of TAP-1 and TAP-18 into TAP-1m and TAP-18m to inactivate their RNA-cleaving activity. Under the same reporting system and growth condition, TAP-1m and TAP-18m failed to grow on the plates with or without theophylline (see also Figure S2), which verified that *E. coli* harboring TAP-1 and TAP-18 survives through RNA self-cleavage after binding with theophylline. We also conducted FAM-labeled *trans*-cleaving experiments to verify that the *in vitro* cleavage efficiency of TAP-1 and TAP-18 were both

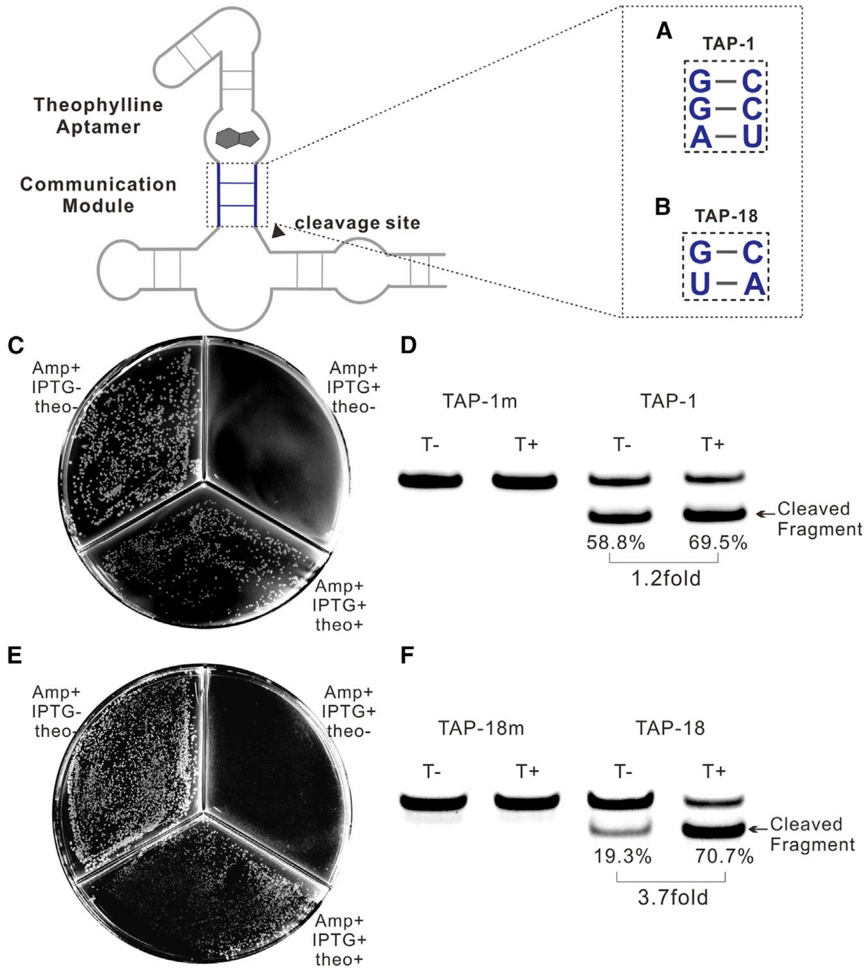

Figure 3. Selection Results

(A) The illustration of CM domain of TAP-1. (B) The illustration of CM domain of TAP-18. (C) The result of different viability for *E. coli* transferred with TAP-1 inserted plasmid on the solid medium added with and without theophylline. The concentration of ampicillin is 100 μg/mL, IPTG is 1 mM, and theophylline is 1 mM. (D) PAGE analysis of FAM-labeled trans-cleaving result of TAP-1. TAP-1m was set as the negative control. The reaction is performed in 50 mM Tris-HCl (pH 7.5) buffer containing 10 mM $MgCl_2$ at 37°C for 1 h with or without 1 mM theophylline. Different fraction of cleaved fragment was identified from the cleaving reactions with and without theophylline (data with error bar was supplemented in Figure S3). (E) The result of different viability for *E. coli* transferred with TAP-18 inserted plasmid on the solid medium added with and without theophylline. The concentration of ampicillin is 100 μg/mL, IPTG is 1 mM, and theophylline is 1 mM. (F) PAGE analysis of FAM-labeled *trans*-cleaving result of TAP-18. TAP-18m was set as the negative control. The reaction is performed in 50 mM Tris-HCl (pH 7.5) buffer containing 10 mM $MgCl_2$ at 37°C for 1 h with or without 1 mM theophylline. Different fraction of cleaved fragment was identified from the cleaving reactions with and without theophylline (quantification data was supplemented in Figure S3).

promoted by theophylline (see also Figure S3). As shown in Figures 3D and 3F, TAP-1 demonstrated 1.2-fold and TAP-18 demonstrated 3.7-fold of promotion in *trans*-cleaving efficiency with 1 mM theophylline in the reaction buffer.

Modulation Efficiency Quantification in Prokaryotic System

The IbsC-based cell survival experiment is hard to quantify intracellular downregulating efficiency of HHAz variants. Therefore, to obtain the accurate off-switching ability of HHAz with and without theophylline adding, the reporting protein IbsC was replaced with a low-background red fluorescent protein *mCherry* (Figure 4A). To verify the feasibility of this strategy, we first inserted HHRz and HHRzm at the 5′ end ORF of *mCherry*. As a result (see also Figure S4), the mRNA with and without self-cleaving ability caused a significant difference in red fluorescence signal, indicating that the self-cleavage of mRNA near 5′ end hinders the translation of *mCherry* and the fusion expression of HHRzm and *mCherry* didn't compromise the red fluorescence signal. Then, TAP-1 and TAP-18 were individually inserted into the vector to fusion express with *mCherry*. As illustrated in Figure 4B, the fluorescent signals of *E. coli* cells containing TAP-1 and TAP-18 revealed significant downregulation in presence of 2 mM theophylline, which is visible to the naked eyes, while the same concentration of the analogs of theophylline such as caffeine, xanthine, hypoxanthine, and 3-methylxanthine cannot cause detectable downregulation of *mCherry*, indicating that both selected HHAz variants maintain the high specificity to theophylline. As TAP-1 afforded much better off-switching efficiency (61%) than TAP-18 (17.5%), we analyzed its responses to different concentrations of theophylline with flow cytometry. The intracellular HHAz TAP-1 was very sensitive to the exogenous regulator and responded significantly toward theophylline from 10 μM to 10 mM, causing the obvious downregulation of *mCherry* protein (see Figure 4C; Figure S4C). Based on the quantitative analysis, the EC_{50} (half-maximal effective concentration) of theophylline for HHAz variant TAP-1 is determined as 212.4 μM (Figure 4D). And the gene-regulation ability of TAP-1 could be greatly affected by introducing mismatches into the communication region of TAP-1(see Figure S5).

Modulation Efficiency in Eukaryotic System

Considering the potential application in future gene therapy, since TAP-1 is obtained in *E. coli*, it is necessary to investigate their modulation ability in the eukaryotic system. Unlike in prokaryotic cells, the HHRz was inserted in the 5′ end of the mRNA of the targeted protein. In eukaryotic cells, HHRz was normally inserted in the 3′ end of mRNA, utilizing the self-cleavage of HHRz causing the loss of 3′ poly(A) tails, which destabilizes the mRNA, leading to the downregulation of *mCherry* protein (see Figure S6B). Thus, HHAz

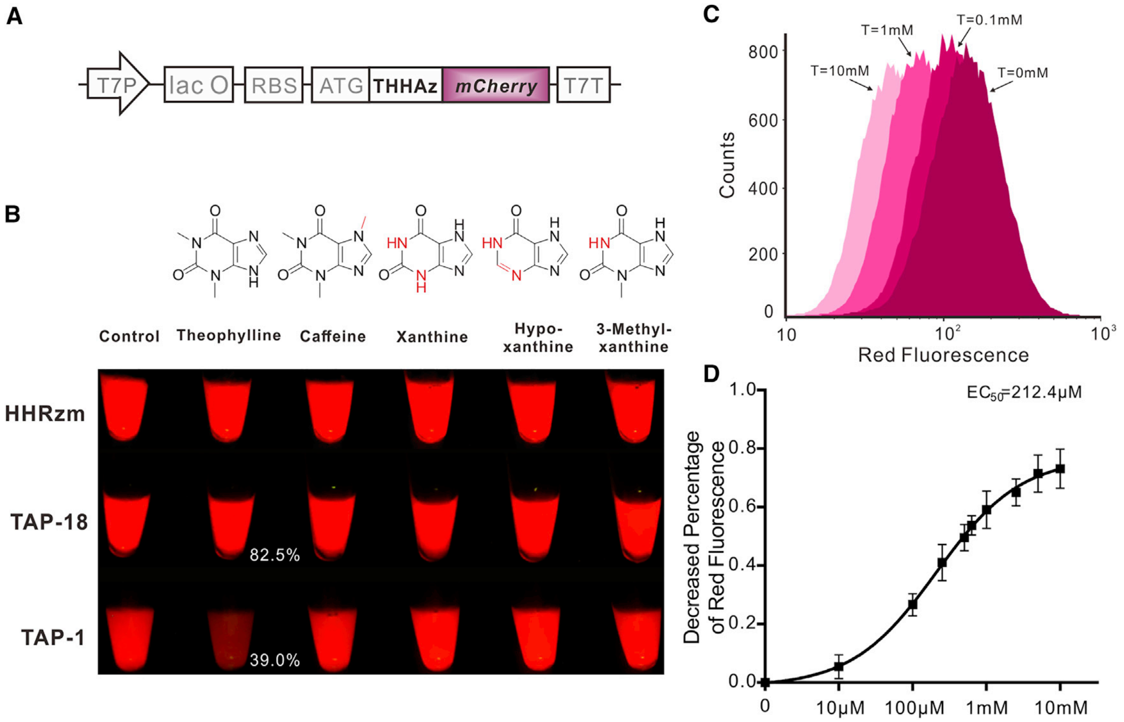

Figure 4. Modulation in Prokaryotic System

(A) The strategy of HHAz fusion expression with mCherry in prokaryotic system. (B) The experiment to investigate the specificity of selected HHAzs. The molecular structure of each analog was illustrated above corresponding tubes. Red line and letters showed the difference from theophylline molecule. The remaining red fluorescence signal after theophylline addition was shown in the picture, and the control samples without adding molecules were set as 100%. (C) Cytometer histograms of red fluorescence signal produced by host cells harboring TAP-1 in reaction of 0 mM, 0.1 mM, 1 mM, and 10 mM theophylline, respectively from right to left. Quantitative data is supplemented in Figure S4C. (D) The decreased percentage of red fluorescence at different concentrations of theophylline were calculated. Decreased percentage of red fluorescence = RT^{-}-RT^{+}/RT^{-}. RT^{-} = red fluorescent intensity without theophylline adding, and RT^{+} = the red fluorescent intensity with theophylline adding. The EC_{50} of TAP-1 is around 212.4 μM. All calculations were made using the GraphPad Prism software (GraphPad Software).

was individually inserted into the 3′ end of exogenous gene *mCherry*, and HEK293T cell was chosen to harbor the constructed vectors. As a result, TAP-1 showed much better gene-regulation results than TAP-18 (see Figure S6C) and maintained its specificity toward theophylline in the eukaryotic system (see Figure S6D).

In recent years, many theophylline-dependent HHAzs have been obtained and verified through different strategies, while no collective comparison has been conducted to figure out which one works in eukaryotic systems with more preferable efficiency. In the work conducted by Hartig et al.,[21] the performance of theo-HHAz,[19] P1F5,[21] and 5.3[21] were evaluated together, and P1F5 showed better performance. Similarly, theo-HHAz,[19] L2bulgeOff2,[11] and MW (not named in the original paper)[22] were evaluated together by Pei et al.,[38] in which MW showed better switching ability. Thus, to conduct a fair comparison and evaluate the performance of TAP-1 under a unified strategy, the chosen HHAz variants, M5,[20] P1F5, MW, H4,[23] and Theo5[17] (shown in Figure 1C) have been inserted at the same position to downregulate the expression of *mCherry*, and HEK293T cell was chosen to harbor the constructed vectors. After transfection and incubation with 1 mM theophylline for 48 h,[17,21,25] all variants downregulated red fluorescence signal effectively. M5 and Theo5, obtained through rational design, individually downregulated 28.6% and 18.7% of the red fluorescence signal. Moreover, MW, theo-H4, P1F5, and TAP-1, obtained through intracellular selection, individually downregulated 15%, 17.7%, 33.6%, and 67.5% of the red fluorescence signal (Figure 5B). Comparing with the quantitative data, the intracellular-selected HHAz variants demonstrated more positive downregulation efficiency than rational-designed ones, suggesting that intracellular selection is more reliable to obtain active HHAz variants to work in the cell.

To take the possible application in future gene therapy into consideration, a lower but safer concentration of theophylline, 0.1 mM, was set to compare the modulation performance of all HHAz candidates. As shown in Figure 5C, except TAP-1 and P1F5, the rest of the HHAz variants showed less than 3% of downregulation efficiency, which indicated that they barely react to the 0.1 mM of theophylline. Moreover, the downregulation efficiency of P1F5 reached 7.6%, which suggested its modest regulation ability toward 0.1 mM of theophylline. However, although the modulation efficiency decreased partly compared to 1 mM of theophylline, TAP-1 afforded a 35.3%

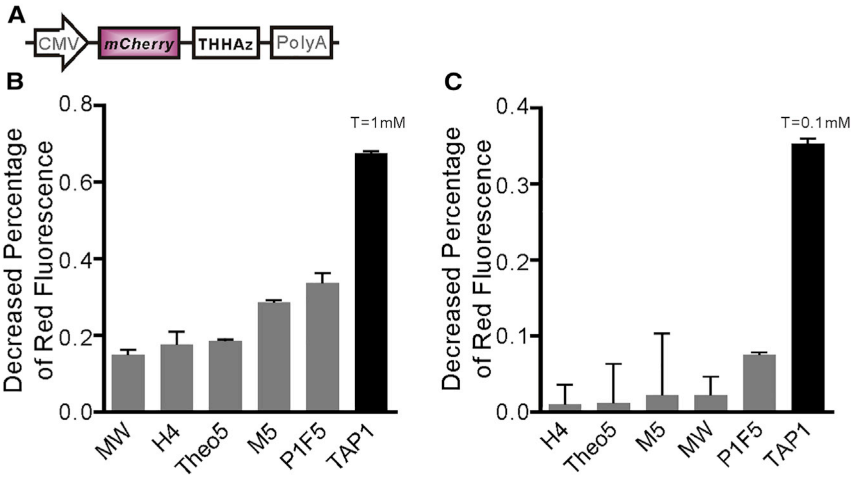

Figure 5. Modulation in Eukaryotic System

(A) The strategy of theophylline-dependent HHAz downregulating mCherry expression in eukaryotic system. (B) Decreased percentage of red fluorescence in HEK293T with 1 mM theophylline. Decreased percentage of red fluorescence = RT^{-}-RT^{+}/RT^{-}. RT^{-} = red fluorescent intensity without 1 mM theophylline adding, and RT^{+} = the red fluorescent intensity with 1 mM theophylline adding. (C) Decreased percentage of red fluorescence in HEK293T with 0.1 mM theophylline. Decreased percentage of red fluorescence = RT^{-}-RT^{+}/RT^{-}. RT^{-} = red fluorescent intensity without 0.1 mM theophylline adding, and RT^{+} = the red fluorescent intensity with 0.1 mM theophylline adding.

decrease of the red fluorescence signal, maintaining its outstanding downregulation ability at 0.1 mM of theophylline. This result demonstrated that TAP-1 can regulate the target gene expression under a rather low concentration of theophylline as 0.1 mM in the eukaryotic system.

DISCUSSION

Without protein recruitment, artificial riboswitch realizes precise gene expression regulation through the specific binding between the RNA and exogenous small molecules, which is convenient for the regulations of transgene expression and the purposeful control of all RNA-related biotechnologies, such as microRNA-mediated RNA interference and CRISPR-mediated base editing. In the perspective of the application, hammerhead ribozyme-based theophylline-dependent aptazyme exhibits unique features. First, hammerhead ribozyme is a promising molecular tool in gene-regulation systems, due to its small size, typically less than 50 base pairs, which could be easily included in gene therapy vectors such as adeno-associated vectors (AVVs) with limited space for additional genes. Second, the theophylline aptamer exhibits particularly specific binding activity toward theophylline, instead of caffeine, which differs from theophylline by only one methyl group. Last but not least, theophylline is a common and safe ingredient in beverages such as tea, so it is possible in the future to modulate exogenous genetic therapy as easy as drinking a cup of tea. Besides, present commonly used methods, including viral vectors[39] or non-viral methods,[40] could be applied in the delivery of TAP-1 based exogenous DNA. As for the targeting of specific cells in gene therapy, tissue-specific promoters[41] in the vector bearing TAP-1 or tissue-specific viruses[42] can be used to transfer the TAP-1 based gene into the targeting cells.

For now, approaches to develop intracellular-applied hammerhead aptazyme are confined with rational design or FACS-based intracellular selection. Besides, the fluorescence-dependent phenotype of single host cells is easily affected by unintentional factors, which makes the selection process more easily influenced by false-positive signals. Here, we developed an intracellular selection strategy basing on a toxin protein *ibsC*, exhibiting several advantages as follows: (1) successfully converting the negative signal, i.e., downregulation of toxin *ibsC*, into a positive phenotype, i.e., colony growth on the solid plates; (2) selecting the HHAz variants with desired property through increasing selection pressure; (3) no dependence on sophisticated equipment. (4) realizing easy-operating and time- and labor-saving selection process. With these features, this proposed method is generally applicable to select HHR-based aptazymes to respond to different ligands, as well as all functional RNA molecules with self-cleaving ability.

With this strategy, a novel theophylline-dependent HHAz variants, TAP-1, have been obtained. After further evaluation of intracellular modulation efficiency with a red fluorescence protein reporting system, TAP-1 showed outstanding switching ability in both prokaryotic and eukaryotic systems with high specificity toward theophylline. Non-negligibly, among presently reported theophylline-dependent HHAz candidates, TAP-1 has been revealed to be equipped with the remarkable modulation range under the variable concentration of theophylline. Last but not least, TAP-1 can effectively regulate gene expression at a much lower concentration of theophylline, 0.1 mM, which is a safe concentration for the human body, indicating that TAP-1 would be a suitable candidate for clinic applications in gene therapy due to its ability to work at low concentration of theophylline. The work of using TAP-1 to regulate the release of therapeutic small interfering RNA (siRNA) molecules the assemble of CRISPR-Cas9-based genome-editing tools is currently underway in our lab.

MATERIALS AND METHODS

Selection Procedure

All nucleotide acids are synthesized from Sangon Biotech (Shanghai, China). The DNA sequence of the randomized pool for CM of HHAz was as follows: 5′-ACGCAGGTACATCCAGCTGATGAGTCCCAA ATAGGACGAAA(N2-5)ATACCAGCCGAAAGGCCCTTGGCAG (N2-5)TCCTGGATTCCACTGCG-3′. N2-5 is 2 to 5 randomized nucleotides in CM. 5′ end of HHRz was restriction enzymes site (BamHI); 3′ end of HHRz was restriction enzymes site (HindIII). The selection dsDNA pools were prepared from fusion PCR products of 3 fragments of DNA sequences. The first fragment is THL3-1, which is consistent. The second fragment, L3R3-6, bears the

randomized sequences. The third fragment has three variants. THL3-2 is the third fragment for L3R3 and L3R6; similarly, THL3-3 is for L3R4 and THL3-4 is for L3R5. This combination is to avoid frame-shift for IbsC (sequence, see Table S1). Fusion PCR was performed as follows: pre-denaturation at 95°C for 1 min, then 20 cycles (30 sec at 95°C, 30 sec at 53°C, 50 sec at 72°C of each cycle).

The selection vector was pT7Oi.[35] DNA products were purified by AxyPrep PCR cleanup kit (Axygen) and double digested by BamHI and HindIII New England Biolabs (NEB) in 37°C for 3 h. Digested fragments of DNA pools were purified by AxyPrep PCR cleanup Kit (Axygen) again. DNA fragments of vector pT7Oi digested by the same restriction enzyme were extracted by AxyPrep DNA Gel Extraction Kit (Axygen). Digested products were ligated by NEB T4 ligase in 4°C overnight. The ligation products were extracted once with equal volume of phenol-chloroform extraction and then ethanol precipitated following recovery in 10 μL sterile deionized water. Electro transformed competent cells were prepared according to the manufacturer's protocol (Bio-Rad) with moderate modifications. Purified ligation products were electro transformed into bacterial expression strain JM109(DE3) (Promega) and recovered in 37°C and 200 rpm for 1 h. All cells were harvested by centrifugation at 4,000 rpm for 5 min following coating on LB plate added with 100 μg/mL ampicillin (Sigma-Aldrich), 1 mM isopropyl β-D-1-thiogalactopyranoside (IPTG; Sigma-Aldrich), and 1 mM theophylline (Macklin, Shanghai, China). In the induction of IPTG, cells with potential active HHAz mutants would survive on the plate. By reapplying the survived colonies on the plates with and without theophylline at same time, it was testified that the different viability was caused by theophylline-modulated HHAz variants. After ensuring the difference viability between plates with and without theophylline adding, mutants were identified by DNA sequencing (Sangon Biotech).

Quantification of Modulation Efficiency Based on Red Fluorescent Protein using FCM

The vector for red fluorescence quantification was the same structure as pT7Oi, only the toxin protein *ibsC* was replaced with red fluorescence protein *mCherry*. Thus, the quantification plasmid was named pT7Om. The recombinant vectors containing WT-HHRz, HHRzm, and selected variants were transformed into bacterial expression strain BL21(DE3) (TransGen, Beijing, China) and recovered in 37°C and 150 rpm for 1 h. 50 μL of transformed cells were coated on LB plates with ampicillin.

More than three monoclones of each structure were inoculated in 1.5 mL LB medium with ampicillin and cultivated in 37°C and 250 rpm until OD600 of culture medium reach 0.3. The cells in logarithmic phase were induced by IPTG (final concentration 1 mM) and divided into two portions. One is added with 1 mM final concentration theophylline; another is added same volume of sterilized water. They were both incubated in 37°C and 150 rpm for 18 h. Then *E. coli* were centrifuged at 4,000 rpm for 5 min and washed with PBS twice to abolish LB medium. Cell pellets were resuspended by PBS and analyzed by FCM (red fluorescence, excitation 561 nm, emission 610 nm). Since off-switching ability is measured on themselves with and without theophylline adding, it was assumed that the two portions of cells that coming from one colony had the similar conditions in their individual cells, as long as theophylline concentration wasn't higher than 1 mM. Accidental errors were ruled out through repeated experiments.

The quantification plasmid used in eukaryotic cells was pmCherry-C1 (a gift from professor Hu Qinxue's laboratory). 2.5 μg/well of the recombinant vectors containing WT-HHRz, HHRzm, and HHAz variants were parallelly transfected with Lipo2000 in Opti-MEM (OMEM, Thermo Fisher Scientific) into HEK293T cells (1.0×10^6 /well) in the 6-well plates. After incubation for 4 h, medium was changed from OMEM into DMEM, supplemented with 10% FBS, penicillin (100 U/mL), streptomycin (100 mg/mL), and 0.1 mM or 1 mM theophylline. After incubation in 37°C and 5% CO_2 for 48 h, cells were washed twice with PBS and collected for FCM analysis.

Specificity Experiment

The recombinant vectors containing HHRzm, TAP-1, and TAP-18 were transformed into bacterial expression strain BL21(DE3) (TransGen) and recovered in 37°C and 150 rpm for 1 h. 50 μL of transformed cells were coated on LB plates with ampicillin. *E. coli* sample harboring the HHRzm, TAP-1, or TAP-18 came from the same colony individually. More than three monoclones of each structure were inoculated in 1.5 mL LB medium with ampicillin and cultivated in 37°C and 250 rpm until OD600 of culture medium reach 0.3. The cells in logarithmic phase were separated into 700 μL each induced by IPTG (final concentration 1 mM), theophylline (1 mM), xanthine (1 mM, Macklin), hypoxanthine (1 mM, Macklin), and 3-methylxanthine (1 mM, Macklin). Each sample was incubated in 37°C and 150 rpm for 18 h. After induction, the OD600 of each sample was tuned with blank LB medium to 1.5. 500 μL of *E. coli* from each sample was centrifuged to remove the LB medium and resuspended with 30 μL sterilized water. After the samples were prepared, red fluorescence signal was directly excited by a real-time blue light monitor (BD-BRV1, Ding Guo Prosperous, Beijing, China) with blue light of 485 nm and the picture was captured with a yellow filter in front of the camera.

As for the eukaryotic specificity experiments, 2.5 μg/well of the recombinant pmCherry-C1 vectors containing TAP-1 parallelly transfected with Lipo2000 in Opti-MEM (OMEM, Thermo Fisher Scientific) into HEK293T cells (2×10^5 /well) in the 24-well plates. After incubation for 4 h, medium was changed from OMEM into DMEM, supplemented with 10% FBS, penicillin (100 U/mL), streptomycin (100 mg/mL), and 0.5 mM theophylline and its analogs individually. After incubation in 37°C and 5% CO_2 for 24 h, cells were washed twice with PBS and collected for FCM analysis.

Extracellular Experiments

As illustrated in Figure S3A, the self-cleaving HHAz variants are split into two parts at Loop1 and Loop2, and the interaction of Loop1 and Loop2 is replaced with totally complementary binding

arms. The binding arms were 8 base pairs each, and S1 is a consistent strand. When the 5 end labeled E binds with S1, and the catalytic core is stabilized, then the cleaving is to be completed with magnesium ion adding. After cleaving, 5 end labeled E is shortened, and the mobility of E is changed, thus the uncleaved E is differentiable with cleaved E on the PAGE gel. Cleavage products were separated on 15% denaturing PAGE and analyzed by Typhoon FLA 7000 IP (GE Healthcare).

5′ FAM-labeled RNA sequences are synthesized from Tsingke (Beijing, China). Constant RNA strands S1 and S1m are *in vitro* transcribed from DNA double strands (synthesized in Sangon, Shanghai, China). DNA oligonucleotides containing the desired mRNA sequences were constructed to imbed T7 promoter sequence at 5′ end. A transcription reaction containing 0.5 mM of DNA template, 10 mM of NTP, T7 RNA polymerase buffer, and 1.25 U/μL T7 RNA polymerase (Thermo Fisher Scientific) were incubated at 37oC for 6 h, followed by ethanol precipitation and purification by 10% PAGE. Sequences of templates were shown in Table S1.

The cleavage reaction of 1 nM FAM-labeled E sequence with 5 nM S1 was carried out in 50 mM Tris-HCl (pH 7.5) buffer containing 10 mM $MgCl_2$ with or without 1 mM theophylline at 37°C. The cleavage reactions were stopped by the addition of 1 volume of stop buffer (80% [v/v] deionized formamide, 50 mM EDTA pH 8.0, 0.025% [w/v] bromophenol blue, 0.025% [w/v] xylene cyanole). Subsequently, the reactions were analyzed with 15% denaturing PAGE. The efficiency of the cleavage reactions was determined by quantitation of the grayscale in the substrates and product bands using a Typhoon Laser scanning imaging system via a phosphor imaging system. The gray gradient was analyzed by ImageQuant TL.

Statistical Analysis

Results were expressed as mean ± SD. All calculations were made using the GraphPad Prism software (GraphPad software).

SUPPLEMENTAL INFORMATION

Supplemental Information can be found online at https://doi.org/10.1016/j.omtn.2020.03.001.

AUTHOR CONTRIBUTIONS

Conceptualization, Z.T.; Methodology, Z.T. and X.H.; Investigation, Q.P. and S.Z.; Formal Analysis, Q.P., S.Z., and Y.Y.; Writing – Original Draft, Q.P.; Writing – Review & Editing, Z.T.; Funding Acquisition, Z.T., X.H., and J.D.; Resources, X.C., F.D., Y.Y., and G.C.; Supervision, Z.T.

CONFLICTS OF INTEREST

The authors declare no competing interests.

ACKNOWLEDGMENTS

This work was supported by the National Natural Science Foundation of China (grant numbers 21572222, 21708037, and 21877108); the Innovative Team of Sichuan Province (grant number 2017TD0021); Sichuan Science and Technology Program (grant numbers 18PTDJ0083 and 2019YFS0331); and China Postdoctoral Science Foundation (grant number 2017M612998).

REFERENCES

1. Winkler, W., Nahvi, A., and Breaker, R.R. (2002). Thiamine derivatives bind messenger RNAs directly to regulate bacterial gene expression. Nature *419*, 952–956.
2. McKeague, M., Wong, R.S., and Smolke, C.D. (2016). Opportunities in the design and application of RNA for gene expression control. Nucleic Acids Res. *44*, 2987–2999.
3. Breaker, R.R. (2011). Prospects for riboswitch discovery and analysis. Mol. Cell *43*, 867–879.
4. Sai Lau, P., and Li, Y. (2011). Functional Nucleic Acids as Molecular Recognition Elements for Small Organic and Biological Molecules. Curr. Org. Chem. *15*, 557–575.
5. Citti, L., and Rainaldi, G. (2005). Synthetic hammerhead ribozymes as therapeutic tools to control disease genes. Curr. Gene Ther. *5*, 11–24.
6. Felletti, M., and Hartig, J.S. (2017). Ligand-dependent ribozymes. Wiley Interdiscip. Rev. RNA *8*, e1395.
7. Famulok, M., and Szostak, J.W. (1992). Stereospecific Recognition of Tryptophan Agarose by in Vitro Selected RNA. J. Am. Chem. Soc. *114*, 3990–3991.
8. Beilstein, K., Wittmann, A., Grez, M., and Suess, B. (2015). Conditional control of mammalian gene expression by tetracycline-dependent hammerhead ribozymes. ACS Synth. Biol. *4*, 526–534.
9. Sack, M., Stifel, J., Kreft, S.G., Deuerling, E., and Hartig, J.S. (2019). Neomycin-dependent hammerhead ribozymes for the direct control of gene expression in Saccharomyces cerevisiae. Methods *161*, 35–40.
10. Li, N., Huang, X., Zou, J., Chen, G., Liu, G., Li, M., Dong, J., Du, F., Cui, X., and Tang, Z. (2018). Evolution of microbial biosensor based on functional RNA through fluorescence-activated cell sorting. Sens. Actuators B Chem. *258*, 550–557.
11. Win, M.N., and Smolke, C.D. (2007). A modular and extensible RNA-based gene-regulatory platform for engineering cellular function. Proc. Natl. Acad. Sci. USA *104*, 14283–14288.
12. Soukup, G.A., and Breaker, R.R. (1999). Engineering precision RNA molecular switches. Proc. Natl. Acad. Sci. USA *96*, 3584–3589.
13. Townshend, B., Kennedy, A.B., Xiang, J.S., and Smolke, C.D. (2015). High-throughput cellular RNA device engineering. Nat. Methods *12*, 989–994.
14. Lee, C.H., Han, S.R., and Lee, S.-W. (2016). Therapeutic Applications of Aptamer-Based Riboswitches. Nucleic Acid Ther. *26*, 44–51.
15. Tang, J., and Breaker, R.R. (1997). Rational design of allosteric ribozymes. Chem. Biol. *4*, 453–459.
16. Nomura, Y., Kumar, D., and Yokobayashi, Y. (2012). Synthetic mammalian riboswitches based on guanine aptazyme. Chem. Commun. (Camb.) *48*, 7215–7217.
17. Zhong, G., Wang, H., Bailey, C.C., Gao, G., and Farzan, M. (2016). Rational design of aptazyme riboswitches for efficient control of gene expression in mammalian cells. eLife *5*, e18858.
18. Liang, J.C., Chang, A.L., Kennedy, A.B., and Smolke, C.D. (2012). A high-throughput, quantitative cell-based screen for efficient tailoring of RNA device activity. Nucleic Acids Res. *40*, e154.
19. Wieland, M., and Hartig, J.S. (2008). Improved aptazyme design and in vivo screening enable riboswitching in bacteria. Angew. Chem. Int. Ed. Engl. *47*, 2604–2607.
20. Kumar, D., An, C.I., and Yokobayashi, Y. (2009). Conditional RNA interference mediated by allosteric ribozyme. J. Am. Chem. Soc. *131*, 13906–13907.
21. Ausländer, S., Ketzer, P., and Hartig, J.S. (2010). A ligand-dependent hammerhead ribozyme switch for controlling mammalian gene expression. Mol. Biosyst. *6*, 807–814.
22. Wieland, M., Ausländer, D., and Fussenegger, M. (2012). Engineering of ribozyme-based riboswitches for mammalian cells. Methods *56*, 351–357.
23. Klauser, B., Atanasov, J., Siewert, L.K., and Hartig, J.S. (2015). Ribozyme-based aminoglycoside switches of gene expression engineered by genetic selection in S. cerevisiae. ACS Synth. Biol. *4*, 516–525.

24. Win, M.N., and Smolke, C.D. (2008). Higher-order cellular information processing with synthetic RNA devices. Science *322*, 456–460.

25. Chen, Y.Y., Jensen, M.C., and Smolke, C.D. (2010). Genetic control of mammalian T-cell proliferation with synthetic RNA regulatory systems. Proc. Natl. Acad. Sci. USA *107*, 8531–8536.

26. Michener, J.K., and Smolke, C.D. (2012). High-throughput enzyme evolution in Saccharomyces cerevisiae using a synthetic RNA switch. Metab. Eng. *14*, 306–316.

27. Tang, W., Hu, J.H., and Liu, D.R. (2017). Aptazyme-embedded guide RNAs enable ligand-responsive genome editing and transcriptional activation. Nat. Commun. *8*, 15939.

28. Ketzer, P., Kaufmann, J.K., Engelhardt, S., Bossow, S., von Kalle, C., Hartig, J.S., Ungerechts, G., and Nettelbeck, D.M. (2014). Artificial riboswitches for gene expression and replication control of DNA and RNA viruses. Proc. Natl. Acad. Sci. USA *111*, E554–E562.

29. Bell, C.L., Yu, D., Smolke, C.D., Geall, A.J., Beard, C.W., and Mason, P.W. (2015). Control of alphavirus-based gene expression using engineered riboswitches. Virology *483*, 302–311.

30. Ketzer, P., Haas, S.F., Engelhardt, S., Hartig, J.S., and Nettelbeck, D.M. (2012). Synthetic riboswitches for external regulation of genes transferred by replication-deficient and oncolytic adenoviruses. Nucleic Acids Res. *40*, e167–e167.

31. Jacobs, M.H., Senior, R.M., and Kessler, G. (1976). Clinical experience with theophylline. Relarionships between dosage, serum concentration, and toxicity. JAMA *235*, 1983–1986.

32. Fozo, E.M., Kawano, M., Fontaine, F., Kaya, Y., Mendieta, K.S., Jones, K.L., Ocampo, A., Rudd, K.E., and Storz, G. (2008). Repression of small toxic protein synthesis by the Sib and OhsC small RNAs. Mol. Microbiol. *70*, 1076–1093.

33. Fozo, E.M., Makarova, K.S., Shabalina, S.A., Yutin, N., Koonin, E.V., and Storz, G. (2010). Abundance of type I toxin-antitoxin systems in bacteria: searches for new candidates and discovery of novel families. Nucleic Acids Res. *38*, 3743–3759.

34. Mok, W.W.K., and Li, Y. (2013). A highly efficient molecular cloning platform that utilises a small bacterial toxin gene. ChemBioChem *14*, 733–738.

35. Huang, X., Zhao, Y., Pu, Q., Liu, G., Peng, Y., Wang, F., Chen, G., Sun, M., Du, F., Dong, J., et al. (2019). Intracellular selection of trans-cleaving hammerhead ribozymes. Nucleic Acids Res. *47*, 2514–2522.

36. De la Peña, M., Gago, S., and Flores, R. (2003). Peripheral regions of natural hammerhead ribozymes greatly increase their self-cleavage activity. EMBO J. *22*, 5561–5570.

37. Khvorova, A., Lescoute, A., Westhof, E., and Jayasena, S.D. (2003). Sequence elements outside the hammerhead ribozyme catalytic core enable intracellular activity. Nat. Struct. Biol. *10*, 708–712.

38. Zhang, Y., Wang, J., Cheng, H., Sun, Y., Liu, M., Wu, Z., and Pei, R. (2017). Conditional control of suicide gene expression in tumor cells with theophylline-responsive ribozyme. Gene Ther. *24*, 84–91.

39. Lukashev, A.N., and Zamyatnin, A.A., Jr. (2016). Viral Vectors for Gene Therapy: Current State and Clinical Perspectives. Biochemistry (Mosc.) *81*, 700–708.

40. Slivac, I., Guay, D., Mangion, M., Champeil, J., and Gaillet, B. (2017). Non-viral nucleic acid delivery methods. Expert Opin. Biol. Ther. *17*, 105–118.

41. Zheng, C., and Baum, B.J. (2008). Evaluation of promoters for use in tissue-specific gene delivery. Methods Mol. Biol. *434*, 205–219.

42. Beatty, M.S., and Curiel, D.T. (2012). Adenovirus strategies for tissue-specific targeting. In Applications of Viruses for Cancer Therapy, D.T. Curiel and P.B. Fisher, eds. (Elsevier Academic Press), pp. 39–67.

OMTN, Volume *20*

Supplemental Information

Intracellular Selection of Theophylline-Sensitive Hammerhead Aptazyme

Qinlin Pu, Shan Zhou, Xin Huang, Yi Yuan, Feng Du, Juan Dong, Gangyi Chen, Xin Cui, and Zhuo Tang

Supplemental Information(SI)

Intracellular Selection of Theophylline-Sensitive Hammerhead Aptazyme

Qinlin Pu[1,2], Shan Zhou[1,2], Xin Huang[1], Yi Yuan[1], Feng Du[1], Juan Dong[1], Gangyi Chen[1], Xin Cui[1] and Zhuo Tang[1,*]

[1]Natural Products Research Center, Chengdu Institution of Biology, Chinese Academy of Science, Chengdu 610041, P.R. China.

[2]University of Chinese Academy of Sciences, Beijing 10049, P.R. China.

[*]Correspondence should be addressed to Zhuo Tang. (tangzhuo@cib.ac.cn). Fax: +86 28 8524 3250; Tel: +86 28 8524 3250

Contents

FigureS1-6

Table S1-2

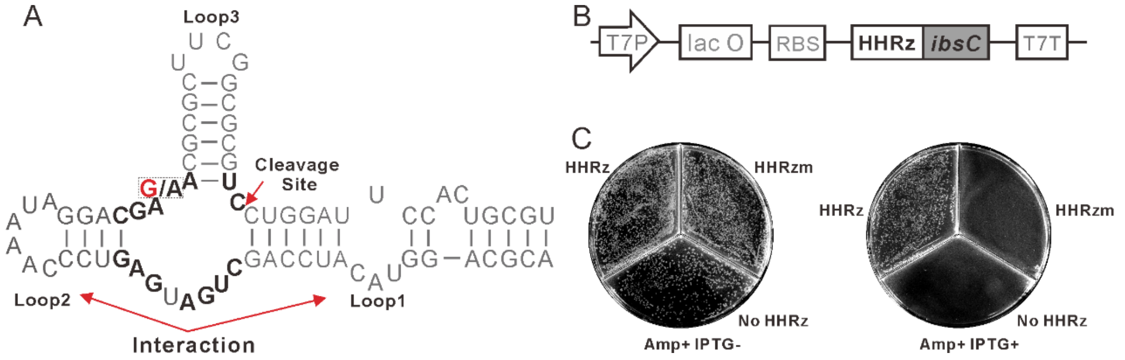

Figure S1:

(A) The illustration of HHRz from *Schistosoma Mansomi*. The black bold letters are the conventional catalytic core of HHRz. HHRz is inactivated when the boxed A mutated into G. Single red arrow shows the self-cleaving site of HHRz. The double red arrow shows the interaction between Loop1 and Loop2, which is the insurance of high self-cleavage efficiency.

(B) The strategy of HHRz fusion expressing with ibsC.

(C) The result of different viability for E.coli transferred with HHR, HHRzm and no HHRz plasmids. Only E.coli transferred with HHRz inserted plasmid survived on the plate with IPTG. The concentration of ampicillin is 100 μg/ml, and IPTG is 1 mM.

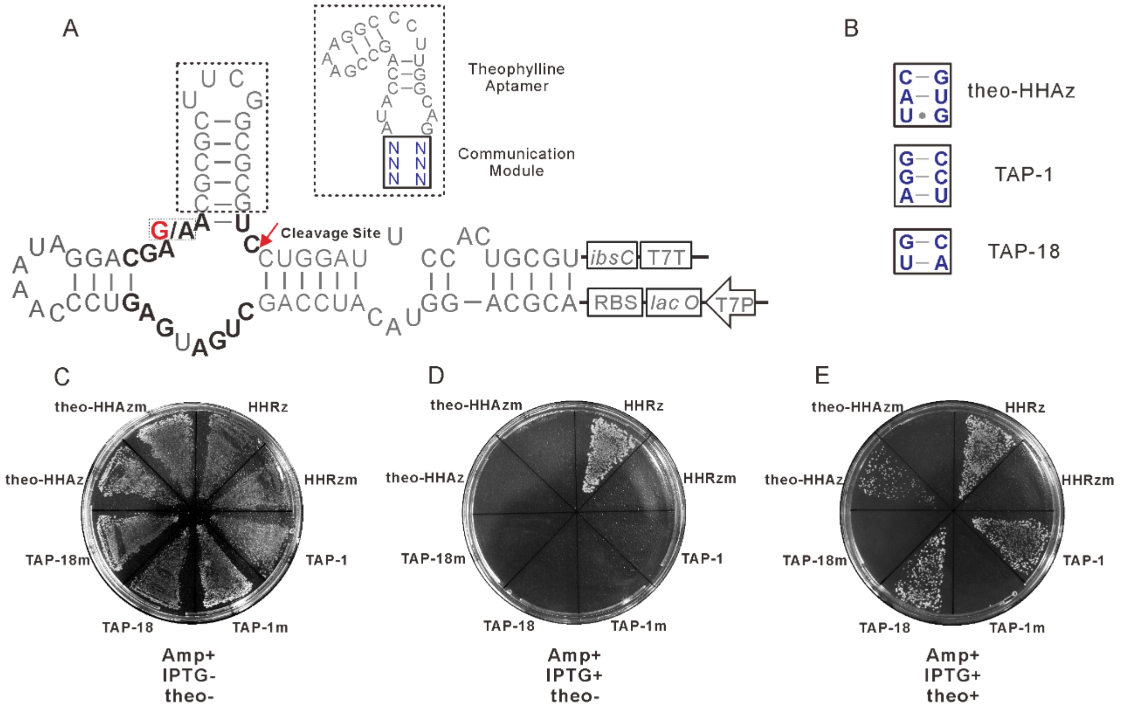

Figure S2:

(A) The strategy of inserting HHRz, HHRzm, TAP-1, TAP-1m, TAP-18, TAP-18m, theo-HHAz, theo-HHAzm individually to fusion express with ibsC. When boxed A14 in catalytic core mutated into G14 in red, the self-cleaving active structures are inactivated.

(B) The illustration of CM domain of TAP-1, TAP-18 and theo-HHAz, and the rest of the nucleotides are the same as illustrated in A. C, D, E. 8 variants were inserted at the same site in the vector, and transferred into JM109DE3 individually at the same time. After recovering in 37 ºC for 1 h, same amount of E.coli was applied on the according area.

(C) The solid medium was added with 100 μg/ml ampicillin.

(D) The solid medium was added with 100 μg/ml ampicillin, 1 mM IPTG.

(E) The solid medium was added with 100 μg/ml ampicillin, 1 mM IPTG and 1 mM theophylline. The comparison between D and E showed that the self-cleavage efficiency was promoted after theophylline adding. When the self-cleaving ability was mutated, no bacterial colony was identified on the plate, referring that the cell survival is dependent on the self-cleavage ability.

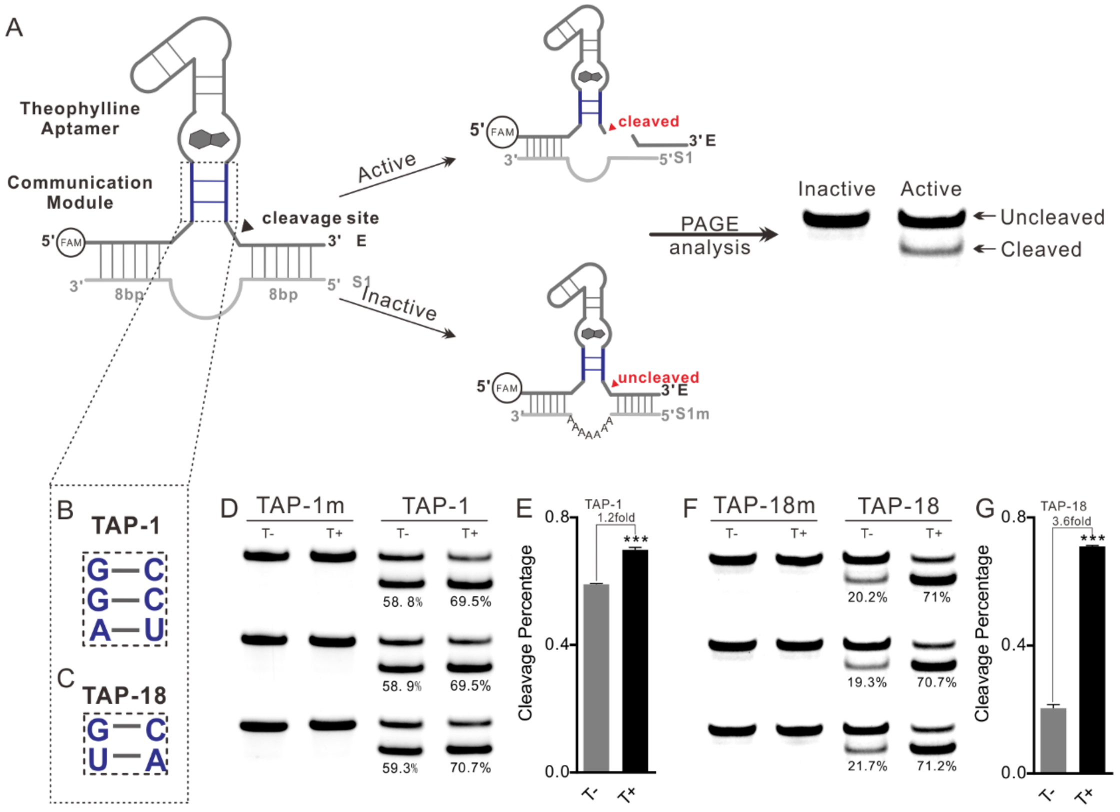

Figure S3:

(A) The illustration of the extracellular cleaving experiments.

(B) The illustration of CM domain of TAP-1, which is consistent with the self-cleaving HHAz variant utilized intracellularly.

(C) The illustration of CM domain of TAP-18, which is consistent with the self-cleaving HHAz variant utilized intracellularly.

(D) Three repeated cleavage reaction of TAP-1 and TAP-1m with or without theophylline adding. The fraction of cleaved TAP-1 were quantified and calculated with ImageQuant TL.

(E) The bar chart of cleavage percentage of TAP-1 with and without theophylline. The cleavage was significantly promoted after theophylline adding. $p<0.001$,***.

(F) Three repeated cleavage reaction of TAP-18 and TAP-18m with or without theophylline adding. The fraction of cleaved TAP-18 were quantified and calculated with ImageQuant TL.

(G) The bar chart of cleavage percentage of TAP-18 with and without theophylline. The cleavage was significantly promoted after theophylline adding. $p<0.001$,***.

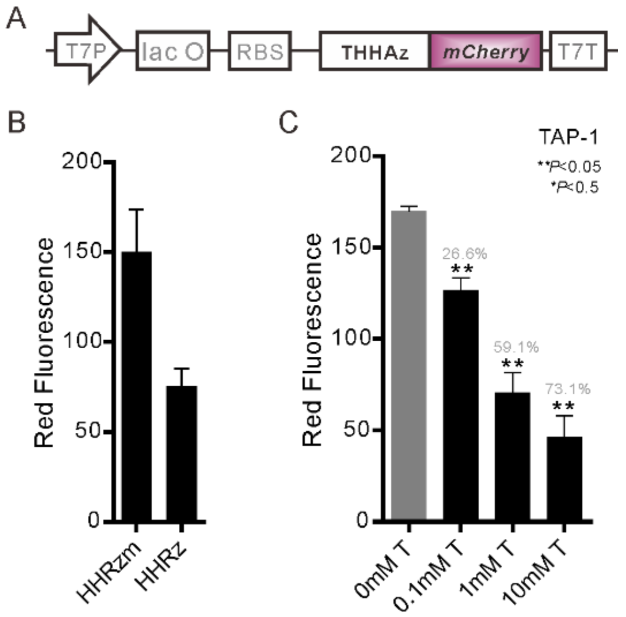

Figure S4:

(A) The strategy of quantifying modulation efficiency of HHAz variants in prokaryotic system. HHRz, HHRzm and HHAz variants are inserted to fusion expression with mCherry. Vectors bearing individual variants were transferred into BL21DE3. After a single colony with individual variants was incubated in LB to reach 0.3 of OD600, they were equally divided into two parts. One of them was added with 1mM of final concentration of IPTG, and another was added with the same amount of IPTG plus 1 mM or 0.1 mM of final concentration of theophylline. Then, both of them were incubated in 37 ºC for 18 h at 150 rpm, and then individual red fluorescence intensity was analyzed through FCM.

(B) The red fluorescence intensity of cells expressing HHRz and HHRzm, verifying the feasibility of the quantifying strategy.

(C) The red fluorescence of cells harboring TAP-1. Three groups of cells were collected at the same time. **, p<0.05; *, p<0.5. All calculations were made using the GraphPad Prism software (GraphPad software, Inc.).

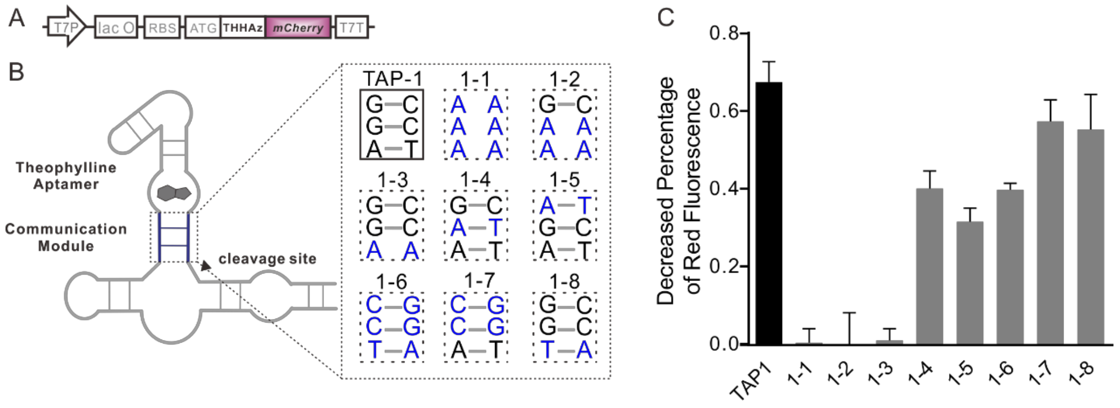

Figure S5:

(A)The construction of modulation strategy in E.coli.

(B)The illustration of designed variants.

(C)The bar chart of modulation percentage of each variants at 1mM of theophylline. Decreased percentage of red fluorescence=RT^{-}-RT^{+}/RT^{-}.

As shown in Figure S5, unmatched base pairs replaced the communication region as 1-1. As expected, 1-1 lost its regulation ability, which testified that the sequence in the communication region formed binding as predicted. Nevertheless, the modulation ability didn't recover as the matched base pairs were recovered one by one as 1-2 and 1-3, which verified that the stability in the communication region is critical for TAP-1. Next, we replaced the G-C pairs individually with slightly weaker binding base pair A-T to improve the flexibility of the communication module for better modulation ability. It turned out that neither variant 1-4 or 1-5 realized improved modulation ability, which indicated that the improved flexibility doesn't help with the modulation ability of TAP-1. Thirdly, as the stability and the flexibility of TAP-1 stayed the same, we examined the sequence specificity by transverse base pairs in the communication module, turning TAP-1 into 1-6, 1-7 and 1-8. Consequently, the regulation ability of the variants is all weakened compared to TAP-1, which verified the sequence specificity of TAP-1 in the communication region. Conclusively, by introducing various mutations into the communication region of TAP-1, it is verified that the specified sequence and stable binding in the communication module ensured its regulation ability.

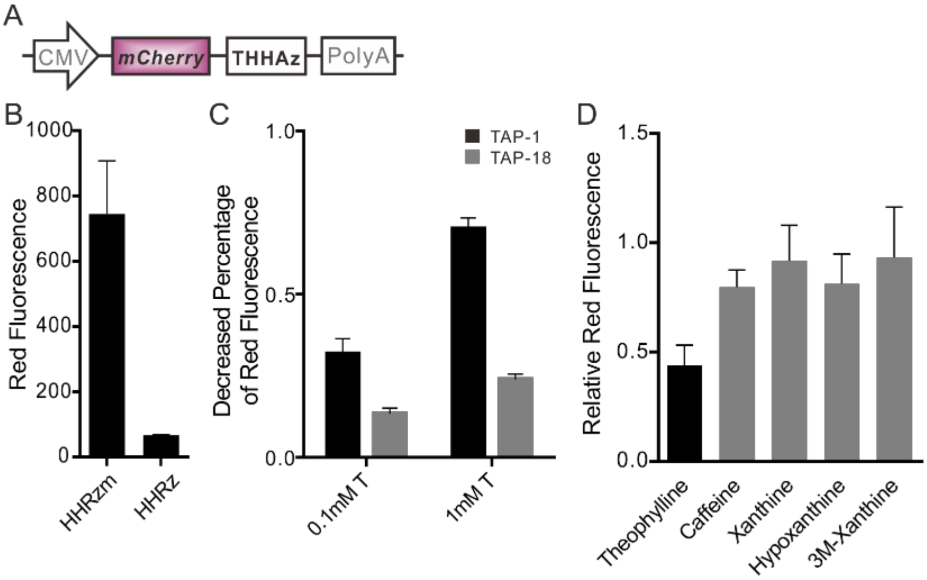

Figure S6:

(A) The strategy of quantifying modulation efficiency of HHAz variants in eukaryotic system. HHRz, HHRzm and HHAz variants are inserted at the 3'end of mCherry.

(B) The red fluorescence intensity of cells expressing HHRz and HHRzm, verifying the feasibility of the quantifying strategy.

(C) The decreased percentage of red fluorescence of TAP-1 and TAP-18 at 0.1mM and 1mM of theophylline. Decreased percentage of red fluorescence=RT^{-}-RT^{+}/RT^{-}.

(D) The relative red fluorescence of TAP-1 in reaction of Theophylline and its analogues. Relative Red Fluorescence=Red Fluorescence with small molecular adding/Red Fluorescence without small molecular adding. Drastically decreased red fluorescence was only observed in the group that was added with theophylline, while the rest groups added with the same concentration of analogues only had slight fluctuations.

Name	Sequences
HHR-F	GCTGGATCCCAGGTACATCCAGCTGATGAGTCCCAAATAGGACGAAACGCGCTTCGG
HHR-R	CGAAAGCTTCGCAGTGGAATCCAGGACGCACCGAAGCGCGTTTCGTCC
HHRm-F	GCTGGATCCCAGGTACATCCAGCTGATGAGTCCCAAATAGGACGAGACGCGCTTCGG
THL3-1	GCTGGATCCACGCAGGTACATCCAGCTGATGAGTCCCAAATAGGACGAAA
L3R3-2	AATAGGACGAAANVATACCAGCCGAAAGGCCCTTGGCAGNNTCCTGGATTCCA
L3R4-2	AATAGGACGAAANVNATACCAGCCGAAAGGCCCTTGGCAGVNNTCCTGGATTCCA
L3R5-2	AATAGGACGAAANVNNATACCAGCCGAAAGGCCCTTGGCAGNNVNTCCTGGATTCCA
L2R6-2	AATAGGACGAAANVNNVATACCAGCCGAAAGGCCCTTGGCAGNVNNNTCCTGGATTCCA
THL3/2-3	CGAAAGCTTGCAGTGGAATCCAGGA
THL3/3-3	CGAAAGCTTCGCAGTGGAATCCAGGA
THL3/4-3	CGAAAGCTTACGCAGTGGAATCCAGGA
theo-HHAz-2	AATAGGACGAAATACATACCAGCCGAAAGGCCCTTGGCAGGTGTCCTGGATTCCA
TAP-1-2	AATAGGACGAAAAGGATACCAGCCGAAAGGCCCTTGGCAGCCTTCCTGGATTCCA
TAP-18-2	AATAGGACGAAATGATACCAGCCGAAAGGCCCTTGGCAGCATCCTGGATTCCA
P1-F5-2	AATAGGACGAAAGCCATACCAGCCGAAAGGCCCTTGGCAGGGTTCCTGGATTCCA
5.3-2	AATAGGACGAAAAATCATACCAGCCGAAAGGCCCTTGGCAGGATTTCCTGGATTCCA
MW-2	AATAGGACGAAACCAGCATACCAGCCGAAAGGCCCTTGGCAGGTTGATCCTGGATTCCA
L2bulgeOff1-1	GCTGGATCCCGAGCTGTCACCGGATG
L2bulgeOff1-2	TGTCACCGGATGTGCTTTCCGGTCTGATGAGTCCGTGTTGCTGATACCAGCATCGTCTG
L2bulgeOff1-3	CGAAAGCTTGAGCTGTTTCGTCCTCGTCCACTGCTGCCAAGGGCATACAGACGATGCTG
M5-2	AATAGGACGAAACACATACCAGCCGAAAGGCCCTTGGCAGTTGTCCTGGATTCCA
3way theoHHAz-1	GCTGGATCCCAGGTACTAGCAGCTGATGAGTCCCAAATAG
3way theoHHAz-2	ATGAGTCCCAAATAGGACGAAACGCCGAATGGCGTCCTGTTCCGATACCAGCCG
3way theoHHAz-3	CGAAAGCTTCCCAGTGGATAGATACTCCGCTGCCAAGGGCCTTTCGGCTGGTATCGGAA
theoH4-2	AATAGGACGAAATGAAAATACCAGCCGAAAGGCCCTTGGCAGTTTAATCCTGGATTCCA
Theo5-2	AATAGGACGAAAGGAATACCAGCCGAAAGGCCCTTGGCAGTCTTCCTGGATTCCA
E-TAP-1	GUAGGGACGAAAAGGAUACCAGCCGAAAGGCCCUUGGCAGCCUUCCUGGAUCC
E-TAP-18	GUAGGGACGAAAUGAUACCAGCCGAAAGGCCCUUGGCAGCAUCCUGGAUCC
S1	CGAAATTAATACGACTCACTATAGGGATCCAGCTGATGAGTCCCTACATC
S1m	CGAAATTAATACGACTCACTATAGGGATCCAGAAAAAAAGTCCCTACATC

Table S1:

The synthesized sequences are listed in this table. The complete sequence of HHRz is obtained by the extension of HHR-F and HHR-R. The complete sequence of HHAz variants are synthesized by fusion PCR of 3 pieces. The THL3-1 is the constant sequence. According to the different length of HHAz variants, the choice of THL3-3 is different. The key point is to combine the 3 pieces of fragments into the multiple of 3 to avoid the frame shift of protein expression.

The selection library is made of 256(2bp), 2304(3bp), 49152(4bp) and 589824(5bp) variants, which is totally no more than 10^6 variants (the randomized area was boxed in the figure below). The selection plasmids were constructed by inserting the corresponding synthesized DNA fragments that contain the randomized communication modules. The corresponding plasmid library was transformed into E. coli cells to construct about 10^7 bacteria library, which covered all variants theoretically.

Name	Sequences	Repitition
theo-HHAz	ACGCAGGTACATCCAGCTGATGAGTCCCAAATAGGACGAAATACATACCAGCCGAAAGGCCCTTGGCAGGGTGTCCTGGATTCCACTGCGT	2
TAP-1	---AGG--------------------------CCT------------------	1
TAP-2	---TAT--------------------------ATA------------------	3
TAP-3	---TG--------------------------CT------------------	2
TAP-4	---TA--------------------------AT------------------	2
TAP-5	---TG--------------------------TT------------------	1
TAP-6	---AA--------------------------TT------------------	1
TAP-7	---AACTA--------------------------CGGTC------------------	1
TAP-8	---TGGTC--------------------------CCTCC------------------	1
TAP-9	---TG--------------------------AT------------------	1
TAP-10	---GGA--------------------------CAT------------------	1
TAP-11	---GC--------------------------GC------------------	1
TAP-12	---TCCCC--------------------------TAGTC------------------	1
TAP-13	---GGA--------------------------ATT------------------	1
TAP-14	---TG--------------------------GT------------------	1
TAP-15	---TGA--------------------------AGC------------------	1
TAP-16	---TGGT--------------------------CCAT------------------	1
TAP-17	---CACT--------------------------AGCG------------------	1
TAP-18	---TG--------------------------CA------------------	1
TAP-19	---TAT--------------------------AAT------------------	1
TAP-20	---TAGCC--------------------------CCACC------------------	1
TAP-21	---GA--------------------------AA------------------	1
TAP-22	---TC--------------------------AT------------------	1
TAP-23	---TCG--------------------------CTT------------------	1
TAP-24	---AGC--------------------------GAT------------------	1
TAP-25	---TA--------------------------GC------------------	1
TAP-26	---CCGC--------------------------TAAC------------------	1
TAP-27	---GC--------------------------CC------------------	1
TAP-28	---CCGGG--------------------------TCCTA------------------	1
TAP-29	---TCG--------------------------GCA------------------	1
TAP-30	---GAGTC--------------------------AGTCG------------------	1
TAP-31	---TG--------------------------CC------------------	1
TAP-32	---TAG--------------------------AAT------------------	1
TAP-33	---ACTCC--------------------------TAGCT------------------	1
TAP-34	---GC--------------------------CA------------------	1
TAP-35	---TC--------------------------TT------------------	1
TAP-36	---GC--------------------------AC------------------	1
TAP-37	---ACCG--------------------------TAGA------------------	1
TAP-38	---TG--------------------------AT------------------	1

Table S2:

The selected sequences are listed in table S2. Theo-HHAz is given the complete sequence as an example. Illustrated in the table are communication module sequences of the variants, and identical sequences in the variants were replaced with dashes. The communication module sequence of theo-HHAz, TAP-1 and TAP-18 were shown in red. Repetition represents the frequency that the variant was identified.
